# Uncertainty-aware automated assessment of the arm impedance with upper-limb exoskeletons

**DOI:** 10.3389/fnbot.2023.1167604

**Published:** 2023-08-24

**Authors:** Samuel Tesfazgi, Ronan Sangouard, Satoshi Endo, Sandra Hirche

**Affiliations:** Chair of Information-oriented Control (ITR), TUM School of Computation, Information and Technology, Technical University of Munich, Munich, Germany

**Keywords:** reliable automated assessment, sensitivity analysis, human-exoskeleton interaction, uncertainty quantification, neuromechanical state estimation, uncertainty-aware simulation

## Abstract

Providing high degree of personalization to a specific need of each patient is invaluable to improve the utility of robot-driven neurorehabilitation. For the desired customization of treatment strategies, precise and reliable estimation of the patient's state becomes important, as it can be used to continuously monitor the patient during training and to document the rehabilitation progress. Wearable robotics have emerged as a valuable tool for this quantitative assessment as the actuation and sensing are performed on the joint level. However, upper-limb exoskeletons introduce various sources of uncertainty, which primarily result from the complex interaction dynamics at the physical interface between the patient and the robotic device. These sources of uncertainty must be considered to ensure the correctness of estimation results when performing the clinical assessment of the patient state. In this work, we analyze these sources of uncertainty and quantify their influence on the estimation of the human arm impedance. We argue that this mitigates the risk of relying on overconfident estimates and promotes more precise computational approaches in robot-based neurorehabilitation.

## 1. Introduction

Medical robotics have advanced greatly with application in many domains, such as robot-assisted surgery (D'Ettorre et al., 2021), service robots in healthcare (Holland et al., 2021) or rehabilitation robotics (Laut et al., 2016). Particularly in the field of physical rehabilitation, an ever-increasing demand for automation technology is observed. Stroke, for instance, is the second leading cause of death worldwide (Feigin et al., 2014) with an increasing trend due to rising life expectancy in many parts of the world (Boehme et al., 2017; Donkor, 2018). However, while stroke is a highly relevant cause for motor impairment, many other neurological disorders, such as cerebral palsy, multiple sclerosis or Parkinsons disease, require similar treatment strategies during rehabilitation to improve or retain motor functions (Krebs et al., 2008). In particular, high-intensity (Ringleb et al., 2008) and repetition training (Kwakkel et al., 1999) have been shown to produce promising recovery results. Due to these requirements, effective rehabilitation is time- and labor-intensive, therefore, both patients and healthcare professionals can benefit greatly from robot-assisted rehabilitation strategies.

In recent years exoskeletons, also referred to as wearable robotic devices (Lo and Xie, 2012), have emerged as a powerful tool for rehabilitation. Since they are designed in a manner that the kinematic chain aligns with the user, sensing and actuation can be performed at the joint level here. One of the main benefits of rehabilitation robotics lies in their application during robot-aided patient assessment. Here, robotic devices are used to monitor patients before. after, or during training, thereby tracking the recovery progress and informing the treatment strategy. In the case of neurological disorders, there are multiple functional impairments, e.g., arm hemiparesis, limited hand dexterity or over-rigid joints, that inhibit motor functions of affected individuals (Carvalho-Pinto and Faria, 2016). Thus, the quantitative estimation of the dynamic parameters underlying these effects using wearable robotic devices can greatly benefit neurorehabilitation. Particularly relevant in the case of stroke is spasticity, a motor disorder described by hyperactivity in tonic stretch reflexes (Mclellan, 1981) which leads muscles to be overly resistive to elongations and thus reduced mobility of the affected limb (Sommerfeld et al., 2004). In current clinical practice, spasticity assessment scales, such as the Modified Ashworth Scale (MAS) are used to evaluate the muscle tone of patients. Here, the clinician induces a passive motion by manually perturbing the target joint of the patient. Concurrently, the muscle tone is assessed by tactually observing the movement resistance. Even though this method has been proven to be useful in clinical practice (Gregson et al., 1999), there are shortcomings that could be alleviated through robotic assessment. Specifically, the coarse and discrete nature of the scales limit the level of precision. Additionally, the evaluation is subjective at its core, which can lead to possibly unreliable and biased estimates that are not consistently reproducible (Blackburn et al., 2002; Raghavan, 2015).

Hence, the deployment of robot-aided assessment is expected to improve the objectivity and repeatability of clinical evaluations (Lambercy et al., 2012). In particular, joint impedance is commonly used as a concise measure for the patient state (Maggioni et al., 2016), since it describes the relationship between joint motion and opposing torque, which is often abnormally increased (Chung et al., 2004). In recent years, a multitude of these assessment approaches based on exoskeletons for upper-limb rehabilitation have emerged. In Ren et al. (2013), an upper-limb exoskeleton quantitatively estimates the joint stiffness of the shoulder, elbow and wrist joints. More recently, a decomposition of the coupled human arm dynamics is proposed to allow the estimation of local and inter-joint stiffness effects following stroke (Zhang et al., 2017). A more extensive impedance estimation is conducted in Wang et al. (2021), where an exoskeleton is used to identify the inertia, viscosity and stiffness components of the elbow joint of patients' with spastic arms using genetic algorithms. Despite the fact that the benefits of robot-aided assessment in comparison to human-administered clinical scales have been demonstrated in studies (Bosecker et al., 2010), exoskeleton applications suffer from the introduction of unintended interaction forces to the user (Jarrassé et al., 2010) with adverse effects on the clinical evaluation. These interaction forces cannot be avoided completely due to uncertainties in the complex physical human-exoskeleton interaction. In particular, sources of uncertainty are known to arise due to kinematic incompatibilities, soft coupling and inaccuracies in the human dynamics model (Pons, 2008). So far, the influence of these sources of uncertainty on the arm impedance estimation has not been analyzed sufficiently, and a quantitative ranking of their impact is missing. However, since the assessment is used to guide the therapy of patients, it is paramount to make these uncertainties explicit in order to increase precision and ensure that clinicians are not misinformed by overconfident assessment results. Therefore, it is important to investigate how uncertain the obtained impedance parameter estimates are and how to effectively reduce uncertainty for exoskeleton-based automated assessment.

### 1.1. Related work

The influence of uncertainties on the robot-aided impedance estimation can be quantified by mean of a sensitivity analysis. These methods investigate how uncertainty in the output of a system, e.g., the result of the automated assessment, is influenced by variations in the input of a system (Pianosi et al., 2016), e.g., sources of uncertainty in the complex human-exoskeleton interaction. Thus, by analyzing these sensitivities and ascribing quantitative measures of importance to each source of uncertainty, the robustness of the automated assessment can be quantified (Thabane et al., 2013). Previously, it has been shown how sensitivity analysis methods are used to support efforts in uncertainty reduction (Hamm et al., 2006) and facilitate robust decision making under uncertainty (Nguyen and de Kok, 2007; Singh et al., 2014).

In general, sensitivity analysis can be approached in multiple ways, with three principle classes identified in Christopher Frey and Patil (2002): analytical, statistical and graphical methods. Typically, analytical methods, such as Kohberger et al. (1978) and Ma et al. (2021), require access to a differential equation model of the system and perform analysis by monitoring the partial derivative over the uncertain parameters (Abraham et al., 2007). In Schiele (2008), an analytical 1 DoF model of the interaction forces induced by kinematic incompatibilities on the elbow joint is proposed. While the presented model was validated experimentally, remaining sources of uncertainty are not considered and it limits the utility of the model as interaction effects cannot be captured by it. Due to the complexity of the human-exoskeleton interaction dynamics, a closed-form description that captures all sources of uncertainty concurrently is not available, which makes analytical sensitivity analysis methods impractical. On the other hand, statistical and graphical approaches solely require access to input-output samples of the system (Christopher Frey and Patil, 2002). Here, samples are generated by evaluating the examined system for a factorial combination of all sources of uncertainty to obtain pertinent statistical information and gain rigorous insights, which is infeasible to do experimentally. Thus, simulations are often used instead (Iooss and Saltelli, 2017). However, to the best of the authors' knowledge, no human-exoskeleton simulation environment considers all of the key sources of uncertainty present during the complex, physical interaction. In Agarwal et al. (2010), for instance, the authors analyzed challenges due to kinematic misalignments on the elbow joint to inform the simulation-based design of an arm exoskeleton. On the other hand, the effect of the human musculoskeletal model on lower-limb exoskeleton control during gait is investigated in Khamar et al. (2019). Lastly, Kühn et al. (2018) present an upper-limb simulation of the human, exoskeleton and their respective coupling where simplified 6 DoF springs are used to model soft-contacts. However, in order to fully understand the effect of uncertainty in exoskeleton-based impedance assessment, all sources of uncertainty and their interaction effects must be considered. Thus, a simulation platform which can systematically express the uncertain human-exoskeleton interaction is required in order to quantify the impact of sources of uncertainty on the estimated impedance parameter.

### 1.2. Contribution

In this work, we perform a sensitivity analysis that quantitatively investigates the influence of various sources of uncertainty on the exoskeleton-based arm impedance estimation. Through this process, a more precise understanding of the uncertainty composition and their prioritization is achieved, which facilitates effective measures to increase the performance of exoskeleton-based automated assessment and reduces the risk of relying on overconfident results. We propose a two-phase approach, where initially the negligible sources of uncertainty are identified, and then a ranking of the most influential factors is performed in the second phase. Due to the complexity of the human-exoskeleton interaction dynamics, we adopt a sampling-based sensitivity analysis which allows us to quantify the influence of each source of uncertainty independently as well as the interaction effects among them. In order to generate the samples required for the analysis, we develop a high-fidelity simulation environment of the human-exoskeleton system that includes the key sources of uncertainty, which are informed by the physical understanding of the system and identified in the literature.

## 2. Materials and methods

**Figure 1:**
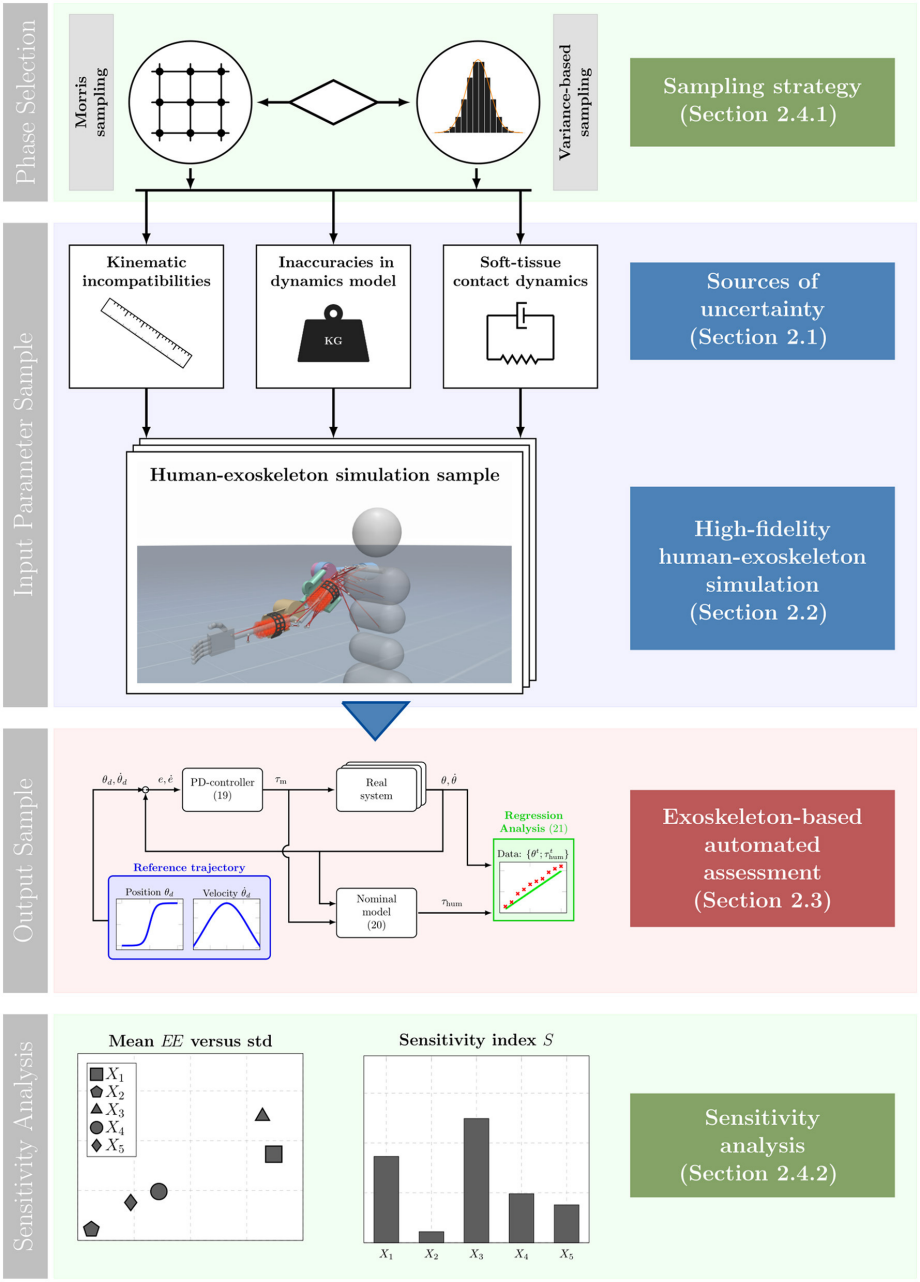
Depiction of the complete, proposed sensitivity analysis scheme. From top to bottom the blocks illustrate the different steps taken during the proposed scheme. First, during the *phase selection* the sampling strategy is determined. Subsequently, in the *input parameter sample* block input samples in the form of human-exoskeleton simulation instances are drawn. The *output sample* block illustrates the generation of output samples using the automated assessment process. Lastly, the input-output samples are used to obtain sensitivity measures which is visualized in the *sensitivity analysis*.

In this section, the technical problem is formulated and the relevant material and methods are shown. An overview of the proposed uncertainty quantification procedure is shown in Figure 1. From top to bottom the colored blocks illustrate the *phase selection*, the process of obtaining *input parameter samples*, the process of obtaining *output samples* and the evaluation procedure using quantitative *sensitivity analysis* methods. First, during the *phase selection* the sampling strategy is determined, which is chosen in accordance to the objective of the respective sensitivity analysis method. Following this, the *input parameter samples* are generated. Here, the examined sources of uncertainty are sampled depending on the previously selected sampling strategy. Then, the input parameter samples are retrieved in the form of parameterized human-exoskeleton simulation instances, where the varied parameters are associated with different sources of uncertainty. Subsequently, the *output sample* block is applied. Here, the exoskeleton-based automated assessment is run for the sampled simulation parameterizations to obtain impedance parameter estimates for the human arm. Finally, the *sensitivity analysis* is performed. Depending on the sampling strategy chosen beforehand, different sensitivity analysis methods are deployed on the estimated impedance parameters to investigate the impact of the modeling uncertainties with respect to the observed estimation error. By deploying this sensitivity analysis scheme we are able to derive the most influential sources of uncertainty that influence the exoskeleton-based arm impedance estimation.

The remainder of the section is structured as follows: in Section 2.1, the dynamics governing the human-exoskeleton system are introduced and a qualitative account on uncertainties in the automated assessment is provided. Subsequently, a high-fidelity simulation of the human-exoskeleton interaction is presented in Section 2.2 with particular focus on including the key sources of uncertainty present in the system. In Section 2.3, the proposed assessment procedure is explained and technical details regarding the estimation process are provided. Finally, in Section 2.4, the deployed sampling strategies and sensitivity analysis methods are presented.

### 2.1. Uncertainty during human-exoskeleton interaction

In order to perform the sensitivity analysis in an interpretable manner it is necessary to have an understanding of the investigated system. To this end, we first formulate the nominal human-exoskeleton interaction model. Subsequently, uncertainties are introduced to the nominal model.

#### 2.1.1. Nominal human-exoskeleton interaction model

The instrumented assessment using an upper-limb exoskeleton is considered in this work. Therefore, we start by establishing the dynamics governing motion of the human arm. We model the dynamics using Euler-Lagrange equations (Featherstone, 2007) of the form,


(1) $$\begin{array}{l} M_{h}\left(q\right)\ddot{q}+C_{h}\left(q,\dot{q}\right)\dot{q}+g_{h}\left(q\right)=\tau_{\text{hum}}+\tau_{\text{int,h}}. \end{array}$$

Here, ***q***∈ℝ^*d*^ is the d-dimensional state vector containing the joint configuration of the human arm, with $\dot{q}\in {\mathbb{R}}^{d}$ describing the angular velocities and $\ddot{q}\in {\mathbb{R}}^{d}$ describing the angular accelerations. On the left side of (1) the matrix $M_{h}:{\mathbb{R}}^{d}\to {\mathbb{R}}^{d\times d}$ denotes the human inertia matrix, $C_{h}:{\mathbb{R}}^{d}\times {\mathbb{R}}^{d}\to {\mathbb{R}}^{d\times d}$ the human Coriolis matrix and $g_{h}:{\mathbb{R}}^{d}\to {\mathbb{R}}^{d}$ the human gravitational component. In addition to the human generated joint torques ***τ***_hum_, an interaction torque ***τ***_int, h_ acts on the human arm, due to the contact with the robotic system. In (1), ***τ***_hum_ represents the projected joint-level torques induced through variations of muscle lengths, muscle activation and the resulting tensions (Shin et al., 2009). Therefore, ***τ***_hum_ describes the summed dynamics of internal origin and contains the relevant joint dynamics parameter necessary to quantify the patient's inner state. In the case of stroke, a viscoelastic model of the human-generated torque during passive mobilization tasks is proposed (McCrea et al., 2003). Thus, we can formulate the human-generated torque ***τ***_hum_ as


(2) $$\begin{array}{l} \tau_{\text{hum}}=K_{h}\left(q,\dot{q}\right)q+D_{h}\left(q,\dot{q}\right)\dot{q}, \end{array}$$

where $K_{h}:{\mathbb{R}}^{d}\times {\mathbb{R}}^{d}\to {\mathbb{R}}^{d\times d}$ and $D_{h}:{\mathbb{R}}^{d}\times {\mathbb{R}}^{d}\to {\mathbb{R}}^{d\times d}$ correspond to the joint stiffness and viscosity matrix, respectively. In McCrea et al. (2003) the validity of linear viscoelasticity parameters for the modeling of resistive torques in personas with chronic stroke is demonstrated. Therefore, it can additionally be assumed that the parameters are independent of the current configuration, which allows the application of standard regression methods. Thus, the instrumented assessment of the patient's state can be reformulated as a linear regression problem using the parametric model


(3) $$\begin{array}{l} \tau_{\text{hum}}=K_{h}q+D_{h}\dot{q}. \end{array}$$

In order to estimate the impedance parameters ***K***_*h*_ and ***D***_*h*_, it is first necessary to extract the human generated torque ***τ***_hum_ in (1). This is not trivial in general, as the intrinsically generated human muscle torque cannot be measured directly. Hence, ***τ***_hum_ has to be inferred using the available measurements and dynamics knowledge. For wearable robots deployed in clinical applications, measurements regarding joint positions and motor torques are typically available (e.g., Trigili et al., 2020). Unless additional expensive and possibly inconvenient force-torque sensors are mounted at the physical interface between human and exoskeleton (An and Hollerbach, 1987), the interaction torque ***τ***_int, h_ is also unknown. To overcome this issue, knowledge regarding the dynamics model of the robotic system can be exploited to replace the unknown interaction torque ***τ***_int, h_. Similar to the human, the exoskeleton is described by its rigid body dynamics


(4) $$\begin{array}{l} M_{e}\left(\theta \right)\ddot{\theta }+C_{e}\left(\theta ,\dot{\theta }\right)\dot{\theta }+g_{e}\left(\theta \right)=\tau_{\text{m}}-\tau_{\text{int,e}}, \end{array}$$

where $M_{e}:{\mathbb{R}}^{n}\to {\mathbb{R}}^{n\times n}$ is the inertia, $C_{e}:{\mathbb{R}}^{n}\times {\mathbb{R}}^{n}\to {\mathbb{R}}^{n\times n}$ the Coriolis matrix and $g_{e}:{\mathbb{R}}^{n}\to {\mathbb{R}}^{n}$ the gravitational component of the exoskeleton dynamics. The joint positions, velocities and accelerations of the robotic system are given by ***θ*** ∈ ℝ^*n*^, $\dot{\theta }\in {\mathbb{R}}^{n}$ and $\ddot{\theta }\in {\mathbb{R}}^{n}$ respectively. In the following, we assume that the kinematic chain of human and exoskeleton align, thereby, resulting in *n* = *d*. Furthermore, the movement of the joints is driven by the motor torques ***τ***_m_ and analogs to (1), an interaction torque ***τ***_int, e_ is exerted on the exoskeleton, which acts in the opposing direction in (4).

In the nominal model, three idealized assumptions are made: first, a perfect alignment of the human and exoskeleton kinematic chain is assumed. Second, no displacement of the attachments occurs during movement. Third, a completely rigid interface transmits forces between the human and exoskeleton. If these assumptions hold, both the human's and exoskeleton's joint kinematics match ***q*** = ***θ*** and the interaction torques can be written to


(5) $$\begin{array}{l} \tau_{\text{int,h}}=\tau_{\text{int,e}}. \end{array}$$

For the sake of the derivation of the nominal model we hypothesize the dynamics of the robotic system and human to be known. Then, it is possible to derive the human generated torque ***τ***_hum_ from (1), (4), and (5):


(6) $$\begin{array}{l} \tau_{\text{hum}}=M_{h}(\theta )\ddot{\theta }+C_{h}(\theta ,\dot{\theta })+g_{h}(\theta )\\ \;+\underset{\tau_{\text{int,h}}}{\underset{︸}{M_{e}(\theta )\ddot{\theta }+\;C_{e}(\theta ,\dot{\theta })+\;g_{e}(\theta )-\tau_{\text{m}}}} \end{array}$$

Since the motor torque ***τ***_m_ and exoskeleton kinematics $\left\{\theta ,\dot{\theta },\ddot{\theta }\right\}$ are measurable and the dynamics are assumed to be known, the human torque ***τ***_hum_, as given in (6), is directly computable. Therefore, all the necessary input and output information are available to estimate the human joint viscoelasticity parameters ***K***_*h*_ and ***D***_*h*_ via linear regression using the parametric model (3):


(7) $$\begin{array}{l} y=X\omega , \end{array}$$

where the labels ***y*** follows from the human torque computation according to (6), the input matrix ***X*** contains the human joint measurements under the assumption that ***q*** = ***θ*** and the viscoelasticity parameters of interest are described by ***ω***. Thereby, performing the regression analysis for each joint yields


(8) $$\begin{array}{l} \underset{y}{\underset{︸}{\left[\begin{array}{c} \tau_{\text{hum},i}^{1}\\ \tau_{\text{hum},i}^{2}\\ \vdots \\ \tau_{\text{hum},i}^{T} \end{array}\right]}}=\underset{X}{\underset{︸}{\left[\begin{array}{cc} q_{i}^{1} & {\dot{q}}_{i}^{1}\\ q_{i}^{2} & {\dot{q}}_{i}^{2}\\ \vdots & \vdots \\ q_{i}^{T} & {\dot{q}}_{i}^{T} \end{array}\right]}}\underset{\omega }{\underset{︸}{\left[\begin{array}{c} k_{h,ii}\\ d_{h,ii} \end{array}\right]}}, \end{array}$$

with ${\left\{\tau_{\text{hum},i}^{t}\right\}}_{t=1}^{T}$ denoting the computed human torques and ${\left\{q_{i}^{t},{\dot{q}}_{i}^{t}\right\}}_{t=1}^{T}$ representing the kinematics measurements of the *i*-th joint at discrete time step *t* over the duration *T* of the assessment. Here, *k*_*ii*_ and *d*_*ii*_ are the *i*-th main diagonal entries of the joint stiffness and viscosity matrices, respectively. The parameter vector ***ω*** can be computed directly given access to inputs ***X*** and labels ***y*** as such:


(9) $$\begin{array}{l} \omega ={\left(X^{⊺}X\right)}^{-1}X^{⊺}y. \end{array}$$

However, while the approach is mathematically convenient and can straight forwardly be implemented, it can result in large estimation errors, because it does not account for the uncertainties in the human-exoskeleton interaction dynamics.

#### 2.1.2. Sources of uncertainty

There are multiple factors that introduce uncertainties to the above described nominal model, which stem from variations in the biomechanics of individuals. In particular three key sources of uncertainty that adversely affect the physical interaction are identified in the literature (Pons, 2008): kinematic incompatibilities, soft contact dynamics and inaccuracies in the nominal dynamics model. In the following these sources of uncertainty and their impact on the nominal dynamics are presented in more detail.

##### 2.1.2.1. Kinematic incompatibilities

**Figure 2:**
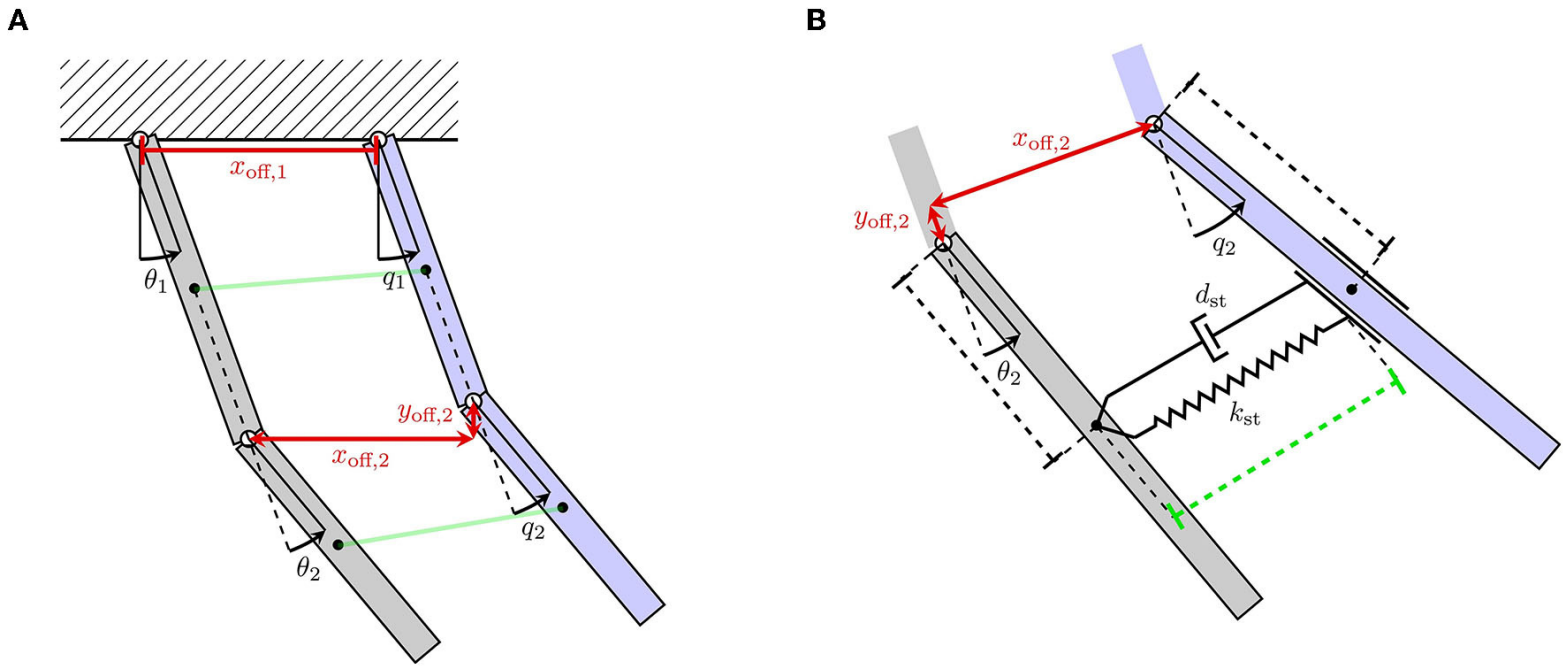
Two-link mechanical model of an interaction between a human (blue) and exoskeleton (gray) arm. **(A)** Illustrates kinematic incompatibilities and the resulting CoR offsets depicted with *x*_off_ and *y*_off_. **(B)** Visualizes soft coupling between the human and exoskeleton link using a Voight-element.

First, we consider kinematic incompatibilities between the exoskeleton and human, which are particularly prevalent in wearable robots with kinematic chains mirroring the human kinematics. These kinematic incompatibilities arise due to anatomical variations between users and variations within a user that occur during motion. Therefore, achieving a perfect alignment is infeasible (Jarrassé and Morel, 2012). Depending on the extent of the mismatch, it is considered a macro-misalignment or a micro-misalignment. Here, macro-misalignments are typically induced by offsets in the center of rotation (CoR) between the human and exoskeleton joints. These CoR offsets are the result of a multiple factors, such as an imprecise donning procedure or translations that occur in the instantaneous center of rotation of human joints for certain movements (Grant, 1973). In Figure 2A, the macro-misalignment due to CoR offsets is shown conceptually for a simplified two-link human-exoskeleton-system moving in the vertical plane. The top and bottom links represent the upper arm and forearm, respectively, emulating motion in flexion/extension direction. Here, the CoR offsets are visualized by *x*_off_ and *y*_off_ using red arrows. While macro-misalignment can be reduced by performing careful donning and including redundant DoFs in the robotic kinematic chain, micro-misalignments still occur despite these mitigation strategies. This is for instance because the human kinematic chain is not comprised of idealized, circular joints. Therefore, misalignments cannot be removed completely in practice and must be explicitly considered for a robust automated assessment.

The main consequence of these kinematic incompatibilities is induced displacements of the attachments between the exoskeleton and human limb during joint motion. Consequently, these displacements result in forces at the physical interface. The resulting impact on the nominal dynamics of the human-exoskeleton interaction can be observed at multiple points. First, the previously assumed joint alignment does not hold anymore, leading to a discrepancy in the joint angles, i.e., ***q*** ≠ ***θ*** in general. Moreover, an offset and joint angle dependent displacement of the attachments along the axial direction occurs, which leads to a change in the interaction torque transmission (5):


(10) $$\begin{array}{l} {\tilde{\tau }}_{\text{int,h}}=B\left(x_{\text{off}},y_{\text{off}},q,\theta \right)\tau_{\text{int,e}}, \end{array}$$

where ***B*** : ℝ^*d*×*d*^ is a *d*-dimensional diagonal matrix with the main diagonal entries describing the displaced attachment points. In (10), ${\overset{\sim }{\tau }}_{\text{int,h}}$ represents the uncertain interaction torques which now depends on the CoR offsets denoted by ***x***_*off*_ and ***y***_*off*_. Similarly, the induced displacement torques depend on the CoR offsets and joint angles deviations (Schiele, 2008). Therefore, we obtain following uncertain human torque under consideration of kinematic incompatibilities:


(11) $$\begin{array}{l} {\tilde{\tau }}_{\text{hum}}=M_{h}(q)\ddot{q}+C_{h}(q,\dot{q})+g_{h}(q)+{\tilde{\tau }}_{\text{int,h}}\left(x_{\text{off}},y_{\text{off}},q,\theta \right)\\ \;+{\tilde{\tau }}_{\text{d}}\left(x_{\text{off}},y_{\text{off}},q,\theta \right), \end{array}$$

where ${\overset{\sim }{\tau }}_{\text{d}}$ denotes the uncertain displacement torques. In addition to ${\overset{\sim }{\tau }}_{\text{int,h}}$ and ${\overset{\sim }{\tau }}_{\text{d}}$, uncertainty also arises in (11) due to the dependence on ***q***, since the human joint angle cannot be measured directly and cannot be inferred accurately from ***θ***, since ***q*** = ***θ*** no longer holds. Note that, given completely rigid bodies, these kinematic incompatibilities would theoretically make movements impossible and lead to extremely high interaction forces, due to the kinematic system being hyperstatic (Jarrassé and Morel, 2012). However, in practice deformation occurs at the physical interface, since the human limb is not rigid, which allows to retain mobility. The uncertainty that arises due to this plasticity is addressed in the following.

##### 2.1.2.2. Soft-tissue contact dynamics

The second important aspect that introduces uncertainty to the physical human-exoskeleton interaction are morphological factors at the coupling between the robot and human. Specifically, the robotic system induces the desired movement by transmitting forces through the soft-tissue of the human limb at the attachment straps. Here, the considered soft-tissue primarily includes muscles, fat tissue and skin, but may also include smaller anatomical parts, such as ligaments, tendons or blood vessels. This is in contrast to the nominal dynamics model which assumes a rigid connection (11). Therefore, the dynamic properties of the human soft-tissue impact the description of the physical interaction.

Soft-tissue is most commonly modeled by elastic or viscoelastic components (Maurel, 1999). Viscoelastic dynamic behavior can for instance be represented by Voight-elements as illustrated in Figure 2B. Here, the soft coupling between the human and exoskeleton link is achieved via a Voight-element at the attachment. Hence, the displacement torques ${\overset{\sim }{\tau }}_{\text{d}}$ and the interaction torque ${\overset{\sim }{\tau }}_{\text{int,h}}$ become functions of the viscoelastic parameters, since all interaction forces are transmitted through soft contacts. It leads to


(12) $$\begin{array}{l} {\tilde{\tau }}_{hum\;}=\;M_{h}(q)\overset{¨}{q}+C_{h}(q,\dot{q})+g_{h}(q)\\ \;+{\overset{˜}{\tau }}_{\text{int},h}(x_{\text{off}},y_{\text{off}},q,\theta ,K_{\text{st}},D_{\text{st}})\\ \;+{\overset{˜}{\tau }}_{\text{d}}(x_{\mathrm{off}},y_{\mathrm{off}},q,\theta ,K_{\text{st}},D_{\text{st}}), \end{array}$$

where ***K***_st_ and ***D***_st_ denote the lumped viscoelastic properties of the coupling due to soft-tissue. In Schiele (2008) a more detailed analysis of the displacement forces and their transmission through soft-tissue modeled as Voight-elements is presented. However, while linear, uniaxial models as shown in (12) are used for practicality, they describe the complex relationship between applied pressure and resulting deformation of the soft-tissue in a simplified manner. A more rigorous approach is to use discrete finite element to approximate the continuous medium and propagating the evolution of the deformation in simulations (Maurel et al., 2002). However, since this is an iterative procedure, it cannot straightforwardly be translated to an analytical model.

##### 2.1.2.3. Inaccuracies in the human dynamics model

Another source of uncertainty that needs to be considered are inaccuracies in the human dynamics model. This is due to significant variations in the biomechanics of each human. To mitigate this, precise measurements of geometrical and inertial properties of the anatomical links are necessary to compute the personalized model parameters required for the human rigid body dynamics (1). However, gathering the information needed to estimate the human model parameter can be expensive, cumbersome and time-intensive (Zajac et al., 2002). Therefore, in clinical practice most commonly standard tables of anthropometric parameters are used (de Leva, 1996) to infer model parameters by scaling the default dynamics model to the height and weight of a particular individual. However, since the approach only yields an approximate measure, uncertainties are introduced. Thus, the uncertain human torque ${\overset{\sim }{\tau }}_{\text{hum}}$ under additional consideration of the modeling inaccuracies is


(13) $$\begin{array}{l} {\tilde{\tau }}_{\text{hum}}=\;{\tilde{M}}_{h}(q)\ddot{q}+{\tilde{C}}_{h}(q,\dot{q})+{\tilde{g}}_{h}(q)\\ \;+{\tilde{\tau }}_{\text{int,h}}\left(x_{\text{off}},y_{\text{off}},q,\theta ,K_{\text{st}},D_{\text{st}}\right)\\ \;+{\tilde{\tau }}_{\text{d}}\left(x_{\text{off}},y_{\text{off}},q,\theta ,K_{\text{st}},D_{\text{st}}\right), \end{array}$$

where ${\overset{\sim }{M}}_{h}$, ${\overset{\sim }{C}}_{h}$, and ${\overset{\sim }{g}}_{h}$ denote the uncertain inertial, Coriolis and gravitational component of the human arm dynamics, which differ from the approximation obtained from the anthropometric tables. We summarize the torque due to the uncertain passive dynamics of the human limb with


(14) $$\begin{array}{l} {\overset{\sim }{\tau }}_{\text{rbd,h}}={\overset{\sim }{M}}_{h}\left(q\right)\ddot{q}+{\overset{\sim }{C}}_{h}\left(q,\dot{q}\right)+{\overset{\sim }{g}}_{h}\left(q\right). \end{array}$$

Thereby, we can write (13) to a more compact form for improved readability


(15) $$\begin{array}{l} {\overset{\sim }{\tau }}_{\text{hum}}={\overset{\sim }{\tau }}_{\text{rbd,h}}+{\overset{\sim }{\tau }}_{\text{int,h}}+{\overset{\sim }{\tau }}_{\text{d}}. \end{array}$$

Here, ${\overset{\sim }{\tau }}_{\text{rbd,h}}$ denotes the uncertain rigid body dynamics of the human arm due to unknown parameters in ${\overset{\sim }{M}}_{h}$, ${\overset{\sim }{C}}_{h}$ and ${\overset{\sim }{g}}_{h}$. Differently to the human limb, the model parameters governing the dynamics of the exoskeleton (4) can reasonably be assumed to be known or can be obtained accurately using classical identification procedures (Hollerbach et al., 2008). Note that in (15), both ${\overset{\sim }{\tau }}_{\text{int,h}}$ and ${\overset{\sim }{\tau }}_{\text{d}}$ are in principle torques that are induced by the interaction with the exoskeleton. However, they differ in the sense that ${\overset{\sim }{\tau }}_{\text{int,h}}$ represents the desired loads that should be transmitted to the human limb, while ${\overset{\sim }{\tau }}_{\text{d}}$ are purely undesired torques due to kinematic incompatibilities. Since the human torque under consideration of uncertainties ${\overset{\sim }{\tau }}_{\text{hum}}$ (15) differs from the nominal human torque ***τ***_hum_ (6) used in the regression analysis (8), errors are introduced to the estimated impedance parameters. In particular, deploying (6) for the computation of the human torque ***τ***_hum_ implicitly allocates torques that are unaccounted for by the nominal dynamics model to be generated due to joint spasticity. Thus, solving the regression problem will not result in the true viscoelasticity parameter ***K***_*h*_ and ***D***_*h*_. By directly comparing the nominal human torque ***τ***_hum_ to the true, uncertain human torque ${\overset{\sim }{\tau }}_{\text{hum}}$, we obtain


(16) $$\begin{array}{l} \underset{y}{\underset{︸}{\tau_{\text{hum}}}}=\underset{\overset{\sim }{y}}{\underset{︸}{{\overset{\sim }{\tau }}_{\text{hum}}}}-\underset{\Delta y}{\underset{︸}{\Delta \tau_{\text{rbd,h}}-\Delta \tau_{\text{int,e}}-{\overset{\sim }{\tau }}_{\text{d}}}}. \end{array}$$

Here, Δ***τ***_rbd, h_ denotes residual torques due to differences in the nominal human dynamics model ***τ***_rbd, h_ and the unknown, true dynamics model ${\overset{\sim }{\tau }}_{\text{rbd,h}}$. Similarly, Δ***τ***_int, e_ represents residual torques due to errors in the interaction torque modeling, while ${\overset{\sim }{\tau }}_{\text{d}}$ are the displacement torques due to kinematic incompatibilities. From (16) it can be seen that the labels ***y*** deployed in (8) do not agree with the true output $\overset{\sim }{y}$, i.e., the human torque ${\overset{\sim }{\tau }}_{\text{hum}}$ under consideration of uncertainties. The difference is summarized in (16) using Δ***y***. Moreover, the measurements for the desired input matrix ***X*** according to (8) are not available, since kinematic incompatibilities result in a mismatch between the human joint angle ***q*** and exoskeleton joint angle ***θ***. Hence, it can be seen how the uncertainties qualitatively influence the outcome of the regression analysis and impact the automated assessment negatively. However, it remains unclear exactly how sensitive the assessment is with respect to the different sources of uncertainty, which we propose to quantify with a sampling-based sensitivity analysis in this work.

### 2.2. High-fidelity human-exoskeleton simulation

In order to perform a sampling-based sensitivity analysis, a highly controlled environment is required. Obtaining the samples experimentally is infeasible, due to the missing ground-truth information and the large sample size that is required. Therefore, in this work we deploy a high-fidelity simulation environment of the human-exoskeleton system to generate samples. To this end, we develop a novel human-exoskeleton simulation which explicitly accounts for the complex contact dynamics present during physical interaction. Here, an optimization-based physics engine called MuJoCo (Todorov et al., 2012) is deployed which is widely used in the modeling of robotic and biomechanical systems in contact-rich environments (Lowrey et al., 2016; Acosta et al., 2022). In particular, three key features of the proposed simulation enable the realistic emulation of the effects caused by sources of uncertainty and thereby facilitate the sampling-based sensitivity analysis: A musculoskeletal model to simulate the human, the consideration of soft contact dynamics at the attachments and a realistic load transmission via a mechanical interface. The proposed human-exoskeleton simulation is shown in Figure 1 in the *input parameter sample* block. Here, the human skeletal system is depicted in gray, while the muscular system is visualized with red lines. Furthermore, the two red cylindrical shapes on the forearm and upper arm represent the simulated human soft-tissue. Also, it can be seen that the physical interface is realized via cuffs and straps that wrap around the human upper and forearm. The complete human-exoskeleton simulation environment is made publicly available.^1^ A brief summary of the key components is presented below. Following this, a more detailed explanation of each of the components of the simulation, their working principles and the performed validations is provided.

**Human musculoskeletal model:** A musculoskeletal model is implemented for the shoulder and elbow. Deploying a musculoskeletal model of the human arm here is necessary for two reasons. First, the simulated muscular system is used to generate the human torque and emulate spastic behavior. Second, the rigid skeletal system facilitates the introduction of variability in the human kinematics and dynamics. Thereby, it is possible to sample over two of the three sources of uncertainty described in Section 2.1.2.
**Soft-tissue simulation:** In the proposed simulation, soft-tissue is explicitly implemented by a composition of multiple micro-elements, which together form an object with viscoelastic material properties. The viscoelastic properties of the soft-tissue object can be varied, thereby allowing to sample over viscoelastic properties of the soft-tissue.
**Physical human-exoskeleton interface:** We simulate the mechanical interface explicitly by implementing cuffs and straps, which enclose the human arm and facilitate a realistic load transmission. Thereby effects that typically arise at the interface, such as attachment displacements, can be emulated.

#### 2.2.1. Simulation of the human musculoskeletal system

A musculoskeletal model is used in the proposed simulation environment. Here, the rigid component of the human arm has five DoFs, three on the shoulder joint and two at the elbow joint. For the shoulder, the human simulation can rotate along the flexion-extension, abduction-adduction and internal-external axis. Regarding the elbow, the simulation allows movement along the flexion-extension and pronation-supination rotations. While a rigid wrist-hand model is also included in the simulation, in our envisioned interaction scenario with the exoskeleton it is not pertinent. The inertial properties of the rigid skeletal system are designed using statistical anthropometric data (Ramachandran et al., 2016) with a default reference person of height 1.75m weighting 70kg. Thereby resulting in a nominal upper arm length of 36.37cm, a nominal forearm length of 34.9cm, a nominal upper arm mass of 2.25kg and a nominal forearm mass of 1.31kg. However, it is possible to adjust all of the parameters to account for variations in the target population.

In addition to the multi-link rigid body dynamics, the simulation accounts for the dynamics induced by the muscular system. In MuJoCo, biological muscles are modeled by means of muscle-tendon systems which induce dynamics dependent on origin and insertion sited and the forces generated by a muscle actuator. Here, the generated muscle force *F*_*m*_ follows the dynamics


(17) $$\begin{array}{l} F_{m}\left(l,v,a\right)=-F_{0}F_{lv}\left(l,v,a\right), \end{array}$$

where *l* is the scaled length of the muscle, *v* is the scaled velocity and *a*∈[0, 1] denotes the muscle activation level. Additionally, *F*_0_ describes the peak active force and *F*_*lv*_ the force-length-velocity function, which are both fitted according to values derived from the experimental findings in Holzbaur et al. (2005). The origin and insertion sites of the muscles are also implemented in accordance with anthropometric data (Ramachandran et al., 2016), thereby ensuring that the dynamics of the simulated musculoskeletal system follow the real-world dynamics closely.

##### 2.2.1.1. Validation of the human musculoskeletal model

In order to check the validity of the simulated human musculoskeletal model, a simulation experiment is performed. Specifically, it is examined whether the moments generated by the muscular system lie in similar ranges as those observed in real experiments. A common clinical procedure to assess the muscle strength is by means of the maximal isometric torque test (Amis et al., 1980; Garcia et al., 2016). Here, we use this procedure to adapt and validate the simulated elbow muscle contraction, which is a useful measure to quantify the neuromuscular properties of spastic muscles (Wang et al., 2019). In the proposed simulation, the dynamics of the elbow are governed by eight muscles. Specifically, four extensor muscles are considered, namely, the long, lateral and median triceps and the anconeus. Moreover, four flexor muscles are regarded, including the long and short biceps, the brachialis and the brachioradialis. The experimental procedure for the isometric torque test in flexion direction is as follows: First, the shoulder is flexed in the sagittal plane at 90deg and mechanically locked in this configuration. While the shoulder is fixed in place, the elbow is flexed in discrete steps of 1deg increments. At each of the discrete increments a maximum contraction of the elbow flexor muscles is applied, and the resulting torque is measured.

**Figure 3:**
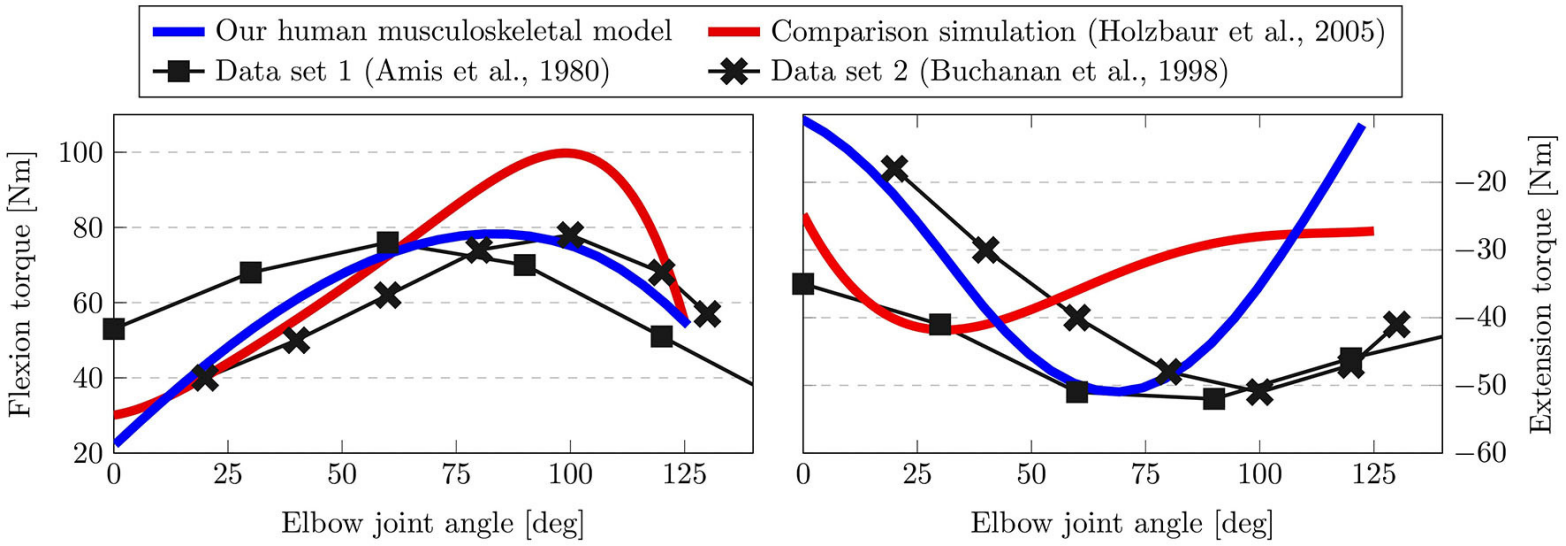
Results of the maximum isometric torque test. Here, the torque generated by the elbow flexors **(left)** and extensors **(right)** is shown over different elbow joint angle. Our human musculoskeletal model (blue) is shown to agree more closely with experimental data than the comparison simulation (red).

The results of performing the maximum isometric torque test in the simulation are shown in Figure 3. Here, the left-hand side shows the isometric flexion torque, while the right side depicts the extension torque. We compare our simulation results (blue) against related biomechanical models of the musculoskeletal system (Holzbaur et al., 2005) (red) and two experimental data sets (Amis et al., 1980; Buchanan et al., 1998). For the isometric flexion torque on the left, it is possible to see that our simulation results match the observed maximum torque of around 80Nm closely, while the comparison simulation exhibits a higher peak at 100Nm. Analogously, our simulation obtains a similar value for the peak extension torque as the experimental data set at −50Nm, while the simulation in Holzbaur et al. (2005) results in a lower absolute value at −41Nm. With respect to the curve shape both data set 1 (Amis et al., 1980) and data set 2 (Buchanan et al., 1998) display different behaviors. This is to be expected due to variability in real experiments and between different subjects, however, the simulation results indicate that our model lies within this range. Particularly, when observing the joint angle at which the peak extension torque is reached for instance, it is clearly visible that our simulation agrees with the experimental data more closely.

#### 2.2.2. Simulation of the upper-limb exoskeleton

In this work, the simulated robotic system is inspired by the specification detailed in Trigili et al. (2020), where an upper-limb exoskeleton with three actuated DoFs on the shoulder level and one actuated DoF for the elbow (flexion-extension) is presented. For the envisioned scenario, we consider all passive and regulatory DoFs to be fixed, therefore, the simulated upper-limb exoskeleton is a four-DoF open chain. Joint friction is implemented via viscous dampers and the inertial properties are designed to roughly match comparable robotic devices. While each joint is associated with an actuator in the simulation, we do not consider elastic actuators here. The actuating motors are also scaled in accordance with the maximum torques the real system can provide according to Trigili et al. (2020). Note that while the simulated exoskeleton is inspired by Trigili et al. (2020), this represents an exemplary device and may be replaced by a different wearable robotic system of interest. The proposed method for the spasticity assessment and sensitivity analysis constitute a general methodology and are therefore not limited to this specific hardware and could be applied to other exoskeleton designs as well.

#### 2.2.3. Physical interface and complex contact dynamics

**Figure 4:**
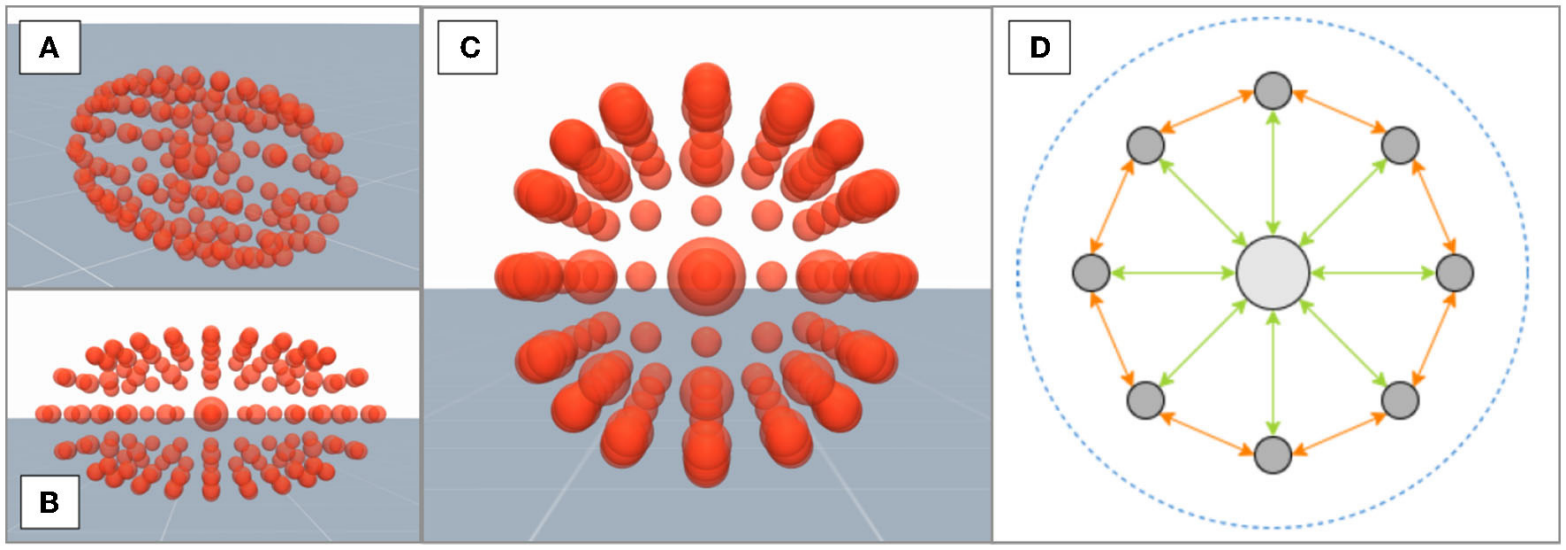
**(A–C)** Depict a composite object with an ellipsoid shape from different viewing angles. **(D)** A cross-section of the composite object with the central element in light gray and external elements in dark gray. Three types of soft constraints hold the elements together: central-external constraints (green), external neighbors constraints (orange), and a global volume constraint (blue).

In our simulation, the physical interface is composed of two contact areas which represent the exoskeleton attachments on the upper and lower arm of the human. On the human side, complexity of the contact dynamics is primarily caused by soft-tissues and their influence on the force transmission at the linkage between the human arm and exoskeleton. In order to replicate the behavior of human soft-tissue in the simulation, three-dimensional composite objects are used, where one central element is surrounded by multiple external elements. Here, the elements of the three-dimensional composite object are arranged such that the resulting geometry approximates the human limb shape and thus a simplification of the commonly used finite element method (Maurel et al., 2002) is achieved. Figures 4A–C depicts the composite object which takes an ellipsoid shape in the simulation environment, where the large sphere at the center of the ellipsoid visualizes the central element of the composite object, while the external elements are illustrated by the smaller spheres. The viscoelastic behavior of the resulting composite object is determined by several soft equality constraints on the relative distance between the different elements, which is illustrated in Figure 4D. Each soft equality constraint generates a force that can be approximately interpreted as a spring-damper link between two elements. Additionally, one constraint acting on all the elements is set to preserve the global volume of the composite object. The parameters of all constraints are fitted to approximate the viscoelastic behavior of real human soft-tissue.

**Figure 5:**
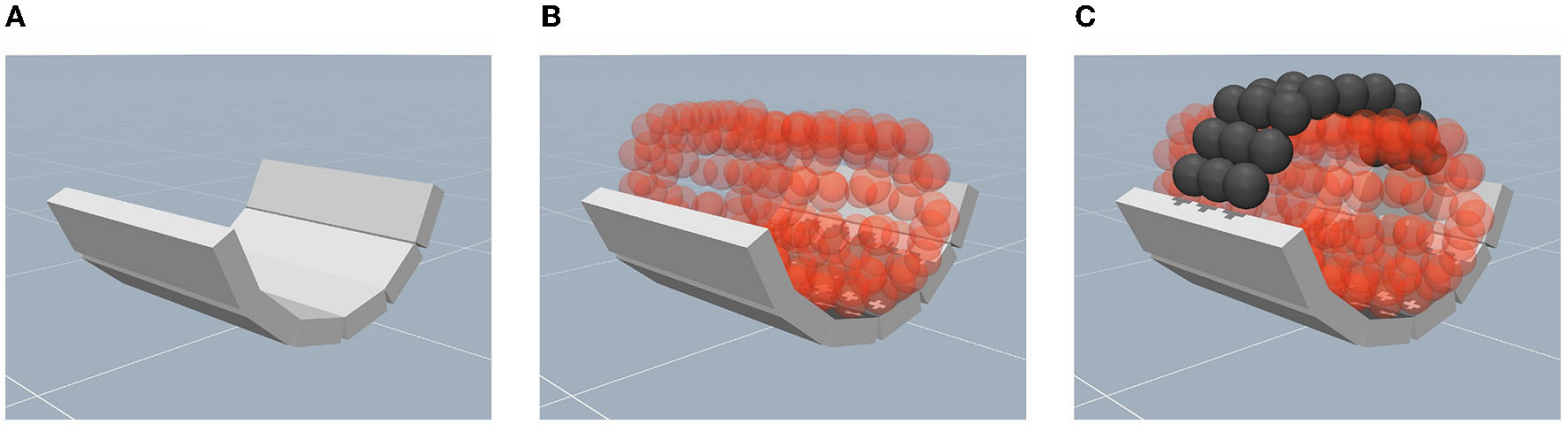
Depiction of the mechanical support at the physical interface in the simulation environment. **(A)** An illustration of the semi-cylindrical cuff composed of welded box primitives. **(B)** An illustration of the placement of the human limb within the cuff. **(C)** The implementation of straps using composite objects to fix the limb to the semi-cylinder.

On the exoskeleton side, forces are generally transmitted to the human arm via the mechanical supports, e.g., cuffs and straps, which induce movement by pushing or pulling the limb (Pons, 2008). Therefore, we follow the same design principle in the simulation in order to render the contact dynamics in high fidelity. First, the arm supports are implemented using a hollow semi-cylinder shape. Since MuJoCo does not directly handle concave bodies, the desired shape is approximately realized by an arrangement of welded box primitives (Figure 5A). Second, the human arm is placed inside the support (Figure 5B). Third, the implementation of the arm straps is realized using composite objects which are arranged in a two-dimensional grid. By welding two opposing sides of the strap to the arm support, the human limb is fixed to the attachment as illustrated in Figure 5C.

##### 2.2.3.1. Validation of the human-exoskeleton contact dynamics

In order to validate the geometric compliance of the simulated limb, the stress-strain relationship of the composite object is investigated in the form of a compression test. In the validation, a uniaxial tension is applied to a solid material and the relationship between compressing stress σ and axial strain ε is quantified (Pelleg, 2012). This property is called Young's modulus *E* and is computed as


(18) $$\begin{array}{l} E=\frac{\sigma }{\varepsilon }=\frac{F/A}{dl/l}, \end{array}$$

where *F* is the applied force, *A* is the unit area and *dl*/*l* is the relative, normalized displacement of the composite body. It characterizes the compressive properties of a material, i.e., a higher Young's modulus *E* describes a stiffer material and a lower *E* indicates a softer material.

**Figure 6:**
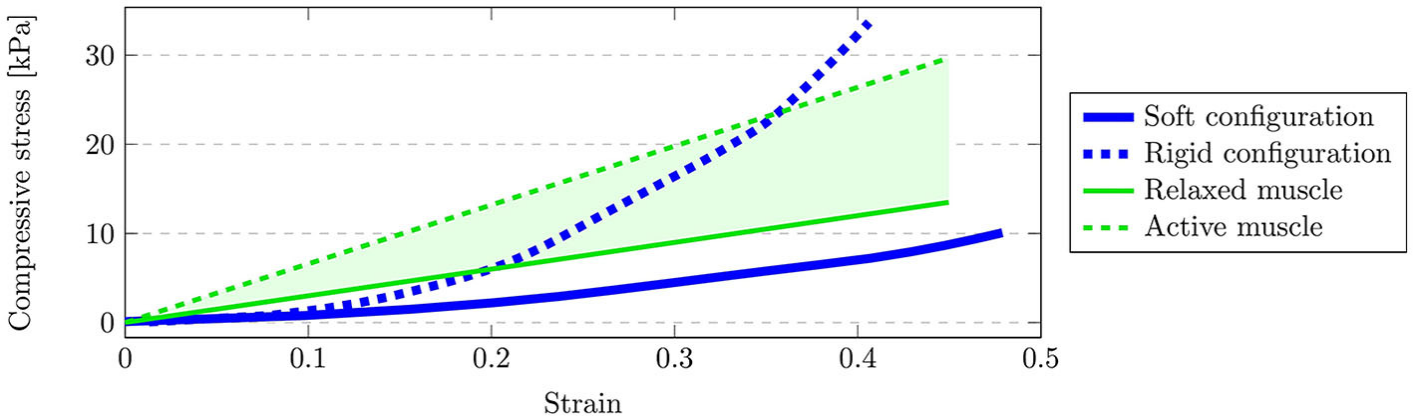
Result of the compression test. The green shaded area depicts the potential range of Young's moduli (Ogneva et al., 2010) determined by relaxed muscles (solid green) and active muscles (dashed green) from experimental data. The range of achievable Young's moduli in the simulation is bound by the soft configuration of the composite object (solid blue) and the rigid configuration (dashed blue).

During the compression test, an incrementally increasing compressive stress is applied to the composite body via two rigid objects to opposing sides of the body. Subsequently, at each incremental step, the Young's modulus was computed from the strain, i.e., the relative deformation, of the composite body. The results are compared with experimental data acquired from mammal muscular tissue (Ogneva et al., 2010) to verify the validity of the simulated soft-tissue. The results of this comparison are shown in Figure 6. Here, the green lines visualize the experimentally determined Young's moduli for relaxed (solid line) and contracted (dashed line) muscle fibers (Ogneva et al., 2010) and the green shaded area indicate the resulting range of potential Young's moduli. Analogously, the blue lines bound the range of achievable Young's moduli via the simulated composite object. The upper and lower bound are obtained by performing the above-described compression test for different parameterizations of the composite object. Given that the simulated, admissible values enclose the experimental data for higher strains, it is possible to approximate the elastic properties of muscle soft-tissue partially. Note however, that the Young's modulus provided from the experimental data (Ogneva et al., 2010) constitutes a linear fit and therefore does not exhibit the typical nonlinear stress-strain relationship which is normally characterized by a region of increasing modulus (Pons, 2008) as depicted by our simulation in Figure 6. Thus, the slight difference for lower strain levels can be explained due to approximation error caused by the linear fit in Ogneva et al. (2010). Furthermore, the experimental data only considers muscle fibers and is therefore expected to vary from the considered soft-tissue, e.g., due to additional fat tissue at the attachments. The additional flexibility in the simulation environment to parameterize lower Young's moduli is thus favorable, since the expected variation generally leads to softer materials.

### 2.3. Exoskeleton-based automated assessment

With the nominal and uncertain dynamics model (Section 2.1) and a human-exoskeleton simulation that includes the key sources of uncertainty (Section 2.2) introduced, the required input samples for the sensitivity analysis can be generated. Here, the input samples are instantiations of the simulation with varying parameters for the different sources of uncertainty. Since we investigate how these uncertainties impact the results of an automated assessment, the output samples are in the form of estimated impedance parameter. The procedure by which these output samples are generated is explained in this section.

**Figure 7:**
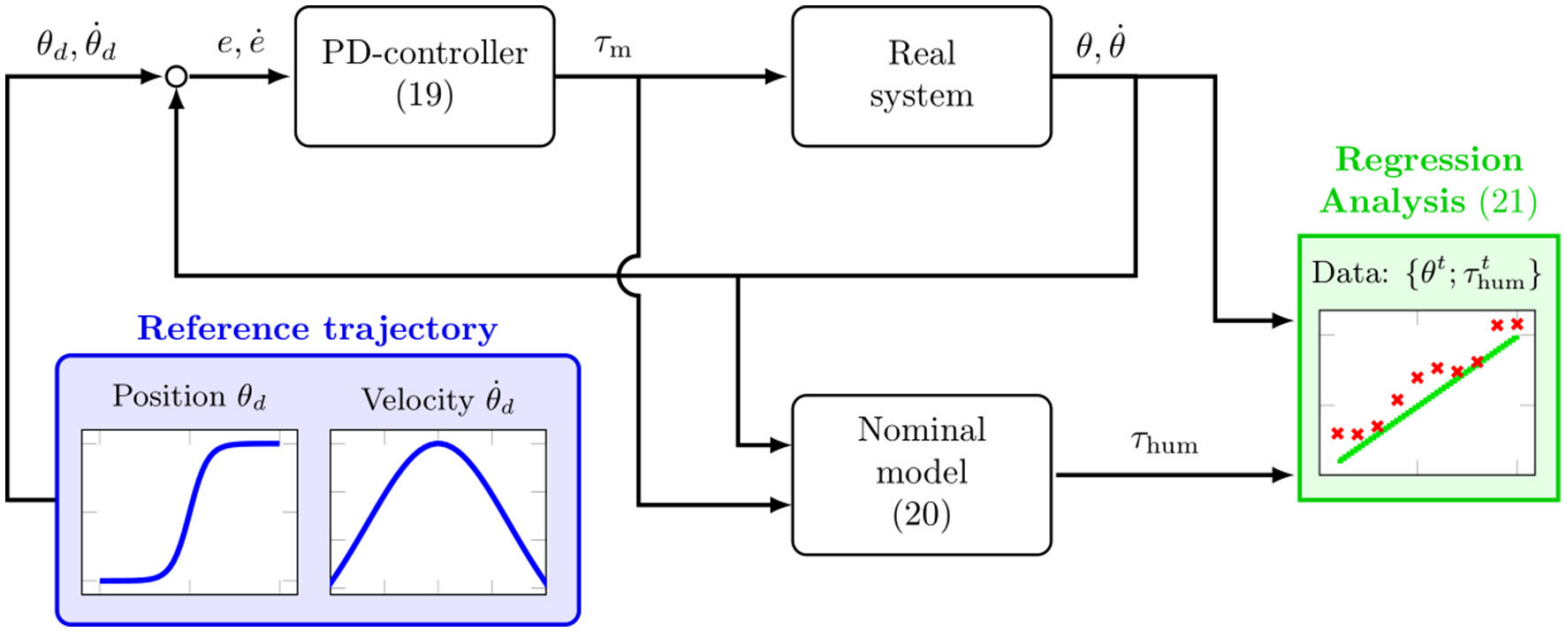
Block diagram of the data collection and estimation scheme for the automated assessment.

In order to perform the spasticity assessment in an automated manner, two components are necessary. First, a data generation procedure is required during which the robotic system interacts with the human arm to induce observations from which the impedance parameters can be inferred. Secondly, the captured data needs to be used to estimate the parameters. In this work, we propose a fully automated scheme for the data generation and estimation that leverages model knowledge to produce the required labels ***y***. The complete scheme is illustrated with a block diagram in Figure 7. Here, the real system represents the true, uncertain human-exoskeleton system which is reproduced in the simulation environment. On the other hand, the nominal model block describes the idealized dynamics model that can be computed analytically. The reference trajectory $\theta_{d},{\dot{\theta }}_{d}$ is depicted in the blue block and is used to observe the joint resistance along a predefined movement, similar to the passive mobilization that is typically performed by a clinician. It acts as an input to the PD-controller, which replicates the manual perturbation generated by the clinician using the exoskeleton.

For the reference trajectory a sigmoid function is selected, since it is known to generate a minimum jerk profile on the joint level (Flash and Hogan, 1985), thus, leading to a natural and comfortable motion for the patient. With the reference trajectory being defined, the exoskeleton applies a torque on the human arm to emulate the manual perturbation performed by the clinician. This is achieved by using the feedback provided by the exoskeleton measurements $\theta ,\dot{\theta }$ and feeding the current tracking error ***e***, ***ė*** into a PD-controller to compute the required motor torque:


(19) $$\begin{array}{l} \tau_{\text{m}}\left(e,ė\right)=K_{p}e+K_{d}\dot{e}, \end{array}$$

where ***e*** = ***θ***_*d*_−***θ*** and ***K***_*p*_, ***K***_*d*_ are the feedback gains of the controller. By applying the motor torque (19), the human-exoskeleton system is moved and, given sufficiently high control gains, the desired trajectory ***θ***_*d*_ is tracked. For the gains of the exoskeleton PD-controller *K*_*p*_ = 50 and *K*_*d*_ = 15 is set. In order to induce spastic behavior in the human simulation, a constant, co-contracting muscle activation of *a* = 0.4 is simulated for the muscles associated with the examined joint. Thereby the human arm will produce a resisting torque opposing the exoskeleton during a change in joint position. The data that is generated during the passive mobilization is used for the regression analysis (8).

For the data generation according to the nominal model, perfect alignment between the human and exoskeleton kinematic chain is assumed. Thus, the measured angles $\theta ,\dot{\theta }$ are assumed to match the human joint kinematics $q,\dot{q}$, thereby providing the nominal input variables ***X*** for the linear regression (8). Furthermore, the output vector ***y*** is required, which comprises measurements of the human internal torque ***τ***_hum_. Since ***τ***_hum_ is not directly measurable, we exploit the nominal model in Figure 7 to overcome this problem. Specifically, using the known motor torque (19) and the nominal dynamics model (6) we can compute the nominal human torque ***τ***_hum_ to be


(20) $$\begin{array}{l} \tau_{\text{hum}}(\theta ,\dot{\theta },e,\dot{e})\;=\;\underset{\tau_{\text{rbd,h}}}{\underset{︸}{M_{h}(\theta )\ddot{\theta }+C_{h}(\theta ,\dot{\theta })+g_{h}(\theta )}}\\ \;+\underset{\tau_{\text{int,h}}}{\underset{︸}{M_{e}(\theta )\ddot{\theta }+C_{e}(\theta ,\dot{\theta })+G_{e}(\theta )-\tau_{\text{m}}(e,\dot{e})}}. \end{array}$$

Here, the parameters of the nominal human model are chosen according to anthropometric data (Ramachandran et al., 2016) with a nominal reference person of height 1.75m and a weight of 70kg, which results in a nominal upper arm length of 33.37cm, a nominal forearm length of 31.9cm, a nominal upper arm mass of 2.25kg and a nominal forearm mass of 1.31kg. Thus, by measuring the trajectory of the exoskeleton joint kinematics $\theta ,\dot{\theta }$ over time and computing the corresponding nominal human torques ***τ***_hum_ according to (20), the regression analysis (8) can be performed for each joint independently.


(21) $$\underset{y}{\underset{︸}{\left[\begin{array}{c} \tau_{\text{hum},i}^{1}\\ \tau_{\text{hum},i}^{2}\\ \vdots \\ \tau_{\text{hum},i}^{T} \end{array}\right]}}\begin{array}{l} =\underset{\hat{X}}{\underset{︸}{\left[\begin{array}{cc} \theta_{i}^{1} & {\dot{\theta }}_{i}^{1}\\ \theta_{i}^{2} & {\dot{\theta }}_{i}^{2}\\ \vdots & \vdots \\ \theta_{i}^{T} & {\dot{\theta }}_{i}^{T} \end{array}\right]}}\underset{\omega }{\underset{︸}{\left[\begin{array}{c} k_{h,ii}\\ d_{h,ii} \end{array}\right]}}, \end{array}$$

where, differently to (8), $\hat{X}$ represent the inputs when the exoskeleton kinematic measurements $\theta ,\dot{\theta }$ are used as a placeholder for the human joint kinematics $q,\dot{q}$. Note that deploying (20) for the computation of the human torques implicitly allocates torques that are unaccounted for by the nominal dynamics model to be generated due to spasticity in the patient's joints. Intuitively, this is analog to the principle applied during manual assessment, where the human limb is assumed to be passive and any encountered resistance is allocated to spasticity. However, as detailed in Section 2.1.2, different sources of uncertainty impact the human-exoskeleton interaction, which result in interaction torques that are not considered in (20). Thus, solving (21) will not result in the true viscoelasticity parameter ***K***_*h*_ and ***D***_*h*_, due to the impact of uncertainties on the regression analysis.

### 2.4. Sensitivity analysis of uncertainties

The goal of this section is to quantify the impact of the uncertainties on the estimated impedance parameters during the exoskeleton-based automated assessment. To this end a sensitivity analysis is performed to examine how variations in the output of a numerical model or simulations can be ascribed to variations of its inputs. We consider uncertainties in the modeling of physical human-exoskeleton interaction as input factors to quantitatively assess their importance. Analogously, the estimated viscoelasticity parameters ***K***_*h*_ and ***D***_*h*_ represent the output samples of the sensitivity analysis. Therefore, sensitivity is defined as the induced variability in the parameter estimates ***K***_*h*_ and ***D***_*h*_ due to variability in the uncertain inputs and is quantified by means of so-called sensitivity indices (Saltelli et al., 2004). Intuitively, these sensitivity indices represent importance measures which are allocated to each input parameter of the simulation, i.e., each source of uncertainty (Pianosi et al., 2016). In this section, the methods used for the sampling-based sensitivity analysis procedure are presented. First, the input sample generation is described in Section 2.4.1. Following this, Section 2.4.2 details the deployed methods for the computation of the sensitivity indices.

#### 2.4.1. Sampling sources of uncertainty

For the input sample generation, we draw samples over different parameterization of the human-exoskeleton simulation. Here, each sampled simulation instance represents a distinct patient with the individual variations present in the population. Six biomechanical parameters are chosen as input factors, where each parameter is associated with a different source of uncertainty. An overview of the parameters, their respective uncertainties and the value ranges is depicted in Table 1. Here, kinematic incompatibilities are produced by varying the length of the human limb. In particular, changes in the upper arm length lead to macro-misalignments and a resultant CoR offset, since the exoskeleton link length remains unchanged. In contrast, varying the human forearm length induces micro-misalignments. The second source of uncertainty investigated during the sensitivity analysis are inaccuracies in the dynamics model. By perturbing the mass of the upper and forearm, errors in the nominal model are evoked as the gravitational component and inertia of the human limb are dependent on the mass. Lastly, uncertainties due to soft-tissue contact dynamics are considered by sampling over different elasticities of the human upper arm and forearm at the attachments. The value ranges of the samples shown in Table 1 are derived from statistical information provided by anthropometric data (Ramachandran et al., 2016). Here, a fixed viscosity of 100Ns/m is chosen for the micro-elements comprising the soft-tissue to avoid numerical instabilities.

**Table 1:** Sources of uncertainty and associated simulation parameters for the input sample generation.

**Uncertainty**	**Simulation parameter**	**Value range**
Kinematic incompatibilities	Length upper arm	27.28cm–37.78cm
Kinematic incompatibilities	Length forearm	28.27cm–34.55cm
Inaccuracies in dynamics model	Mass upper arm	0.3kg–3.41kg
Inaccuracies in dynamics model	Mass forearm	0.1kg–1.82kg
Soft contact dynamics	Elasticity upper arm	100.5N/m–974.43N/m
Soft contact dynamics	Elasticity forearm	100.5N/m–974.43N/m

In addition to defining the input variability space, i.e., the value ranges shown in Table 1, further design choices regarding the sampling strategy have to be made. In general two classes of sampling concepts can be differentiated, One-At-a-Time (OAT) and All-At-a-Time (AAT) methods (Pianosi et al., 2016). While in OAT methods variations are induced by perturbing one input parameter only and keeping all other fixed, AAT methods induce output variations by varying all input parameters concurrently. The main advantage of OAT in comparison to AAT sampling is the reduced computational load due to fewer samples being required. However, because of the concurrent sampling in AAT methods, the joint influence of input factors due to interaction between the parameters can be analyzed, thereby, providing more insights (Pianosi et al., 2016). Depending on the deployed method to estimate the importance measures, both approaches can be beneficial. Therefore, the following section presents sensitivity analysis methods with distinct sampling strategies for different investigation purposes.

#### 2.4.2. Sensitivity analysis methods

Depending on the setting and purpose of the sensitivity analysis, different methods are appropriate. In Saltelli et al. (2008) two main purposes are introduced. First, the goal of ranking the most relevant input factors which is called *factor prioritization*. Second, identifying input factors with negligible impact which is called *factor fixing*. Beyond these two main settings, other purposes are introduced as well. However, given that the proposed sensitivity analysis is supposed to inform the decision making process in clinical practice and lead to more robust spasticity assessment, our quantitative analysis is mainly focused on factor prioritization and factor fixing, since these information lead to a practical guide to performing more robust automated assessment. Additional information may also be derived by qualitative sensitivity analysis methods, e.g., using scatter plots (Beven, 1993; Kleijnen and Helton, 1999).

Furthermore, potential interactions between the investigated sources of uncertainty should also be considered. Since these interactions may emerge for various parameters and it is a-priori unknown how the interactions behave with respect to the magnitude of the parameters, we ideally want to perform a dense sampling over the input variability space. To this end global sensitivity analysis methods are preferred, which investigate variations over the complete range of admissible inputs. Global sensitivity analysis methods have previously been shown to facilitate tasks such as supporting efforts in uncertainty reduction (Hamm et al., 2006) and facilitating robust decision making (Nguyen and de Kok, 2007; Singh et al., 2014).

##### 2.4.2.1. Elementary effects method

Given these requirements, there are multiple viable sensitivity analysis methods. First, Morris method (Morris, 1991), also referred to as *elementary effects test*, is an efficient and suitable approach to perform factor prioritization and fixing. Here, a perturbation-based design is deployed, where the whole input space is explored by applying perturbations to each input factor separately and computing global sensitivity measures from the probed samples. This is done by computing so-called elementary effects *EE* for each input factor *x*_*i*_


(22) $$\begin{array}{l} EE_{i}=\frac{f\left(x_{1},\ldots ,x_{i-1},x_{i}+\Delta_{i},x_{i+1},\ldots x_{K}\right)-f\left(x_{1},\ldots ,x_{K}\right)}{\Delta_{i}}, \end{array}$$

where ***x*** = (*x*_1_, *x*_2_, …, *x*_*K*_) represents a set of input parameters, *f*(***x***) denotes the function that maps inputs to model responses, *K* is the total amount of examined input parameters and Δ_*i*_ is the perturbation applied to the *i*-th input parameter. In order to achieve a global measure of sensitivity, the input space is sampled with *r* trajectories, each consisting of *K*+1 sampling points, where each point differs in just one input factor by a fixed amount Δ (Morris, 1991). Thereby, each trajectory allows for the computation of one *EE* per input factor and the sensitivity measures for each parameter can be computed as such:


(23) $$\begin{array}{l} \mu_{i}=\frac{1}{r}\sum_{j=1}^{r}EE_{i}^{j}\\ \;=\frac{1}{r}\sum_{j=1}^{r}\frac{f(x_{1}^{j},\ldots ,x_{i}^{j}+\Delta_{i}^{j},\ldots x_{K}^{j})-f(x_{1}^{j},\ldots ,x_{K}^{j})}{\Delta_{i}^{j}} \end{array}$$

(24) $$\begin{array}{ll} \sigma_{i}^{2} & =\frac{1}{r-1}\overset{r}{\underset{j=1}{\sum }}{\left(EE_{i}^{j}-\mu_{i}\right)}^{2}, \end{array}$$

where $\Delta_{i}^{j}$ represents the perturbation of the *i*-th input parameter $x_{i}^{j}$ in trajectory *j* and $EE_{i}^{j}$ denotes the computed elementary effect associated with parameter *x*_*i*_ along trajectory *j*. Here, the mean μ and standard deviation σ of the elementary effects *EE* are proposed as sensitivity measures (Saltelli et al., 2008). In particular, μ (23) represents how much the input parameter affects the output, while σ (24) is a measure for the induced effects due to interaction with other inputs, i.e., how much *EE*_*i*_ varies when changes in the remaining *i*−1 parameters occur. Specifically, a small σ_*i*_ implies that the effect of parameter *x*_*i*_ on the output, which is shown by μ_*i*_, is independent of the other parameters. Therefore, Morris method is particularly well suited for factor fixing, since a simultaneous consideration of both μ and σ allows the identification of negligible input factors, which have both little interaction with the other inputs (small σ) and do not influence the output strongly (small μ). Moreover, applying this approach requires relatively few samples, which further increases its utility for factor fixing in cases where model evaluations are expensive. However, since it is a perturbation-based OAT method, it may lead to erroneous results if the target system exhibits high-frequencies in its response to variations in the input (Pianosi et al., 2016).

##### 2.4.2.2. Variance-based sensitivity analysis

An alternative approach that facilitates the analysis of output sensitivity with respect to each input factor over their complete value range are variance-based sensitivity analysis methods, also referred to as Sobol method (Sobol, 1993). Here, modeling uncertainty is specifically considered by regarding the input parameters as stochastic variables with a defined probability distribution. Thereby, a conceptual link between sensitivity and uncertainty is exploited and sensitivity is analyzed by investigating how uncertainty in the input propagates to the output variables. Subsequently, the relative contribution of each input is decomposed and used as a measure of sensitivity. To this end variance is used as a measure to quantify uncertainty. The so-called *first-order effect* *S*_*i*_, which is a measure for the individual contributions of inputs to the output variance, is computed as


(25) $$\begin{array}{l} S_{i}=\frac{𝕍(z)-{𝔼}_{x_{i}}\left[{𝕍}_{x_{-i}}(z|x_{i})\right]}{𝕍(z)}, \end{array}$$

where *z* = *f*(***x***) is the output variable, 𝔼 denotes the expectation and 𝕍 the variance. Here, 𝕍_*x*_−*i*__(*z*∣*x*_*i*_) expresses the conditional variance of the output *z* over *x*_−*i*_, i.e., all inputs except *x*_*i*_, given that *x*_*i*_ is fixed. Analogously, 𝔼_*x*_*i*__(*z*∣*x*_*i*_) denotes the conditional expected value. Therefore, the second term in (25) expresses the expected variance in the output given that the *i*-th input *x*_*i*_ is fixed. A small value for this expectation, and consequently a high value for *S*_*i*_, implies that a significant reduction in output variance can be achieved by fixing *x*_*i*_ (Saltelli et al., 2008). Thus, the first-order index *S*_*i*_ is a measure for the direct contribution of an input to the output variance, which in turn functions as a place-holder for sensitivity.

On the other hand, the *total-order index* *S*_*Ti*_ indicates the total effect of an input *x*_*i*_ on the output variance including interactions with other input factors (Homma and Saltelli, 1996) and is defined as


(26) $$\begin{array}{l} S_{Ti}=\frac{{𝔼}_{x_{-i}}\left[{𝕍}_{x_{i}}(z|x_{i})\right]}{𝕍(z)}. \end{array}$$

Moreover, variance-based methods allow for the computation of further, higher-order indices, such as second-order or third-order ones. Thereby, by computing all 2^*K*^−1 orders, variance-based sensitivity measures can theoretically capture the sensitivities present in the system completely. However, since this is computationally infeasible in practice, a good approximation can be achieved by computing only the first-order and total-order terms (Saltelli et al., 2004).

Thus, variance-based methods are well equipped to analyze sensitivities in a principled manner by both quantifying the importance of individual inputs and groups of inputs. Moreover, an uncertainty-aware modeling paradigm is supported and, by sampling the input space using probability distributions, the full range of input variations can be investigated. However, due to their sampling-intensive nature, it is impractical to deploy them directly when model evaluations are expensive. Therefore, we propose to use both the elementary effect test and variance-based sensitivity analysis in conjunction. Thereby, non-influential input parameters are detected by the efficient elementary effect method and can be discarded prior to performing a more extensive analysis using variance-based methods.

## 3. Results

In this section we present the findings of performing the proposed two-phase sensitivity analysis scheme. First, in Section 3.1 the elementary effect test is deployed to screen parameters that do not effect the automated assessment outcome significantly and can therefore be fixed for subsequent investigations. Second, the variance-based sensitivity analysis is performed on the remaining input parameter in Section 3.2 to determine the relative importance of the different model uncertainties. Lastly, a qualitative analysis of the obtained samples is conducted in Section 3.3 to provide further insights. For clarity of presentation the automated assessment is limited to the estimation of the elbow joint stiffness. The presented sensitivity analysis is implemented in Matlab using the SAFE toolbox (Pianosi et al., 2015), while the simulation model is implemented in Python using the MuJoCo physics engine (Todorov et al., 2012).

### 3.1. Factor fixing using elementary effects

In order to identify non-influential parameters, we deploy the elementary effect method as described in Section 2.4.2. To this end, input parameter samples are drawn for which the human-exoskeleton simulation is instantiated and subsequently the automated assessment is run for each model instance to generate the respective output samples. Here, we use a radial design for sampling the input parameter hyperspace, since it has been shown to achieve superior performance for computing elementary effects (Campolongo et al., 2011). A total of *r* = 150 trajectories is generated for *k* = 6 input parameters, which are listed in Table 1, resulting in 1050 sampling points. For the generation of the random sampling vectors required in the radial design, the well-established Latin hypercube approach (McKay et al., 1979; Helton and Davis, 2003) is used. Moreover, a uniform distribution of the input parameter space is assumed.

**Figure 8:**
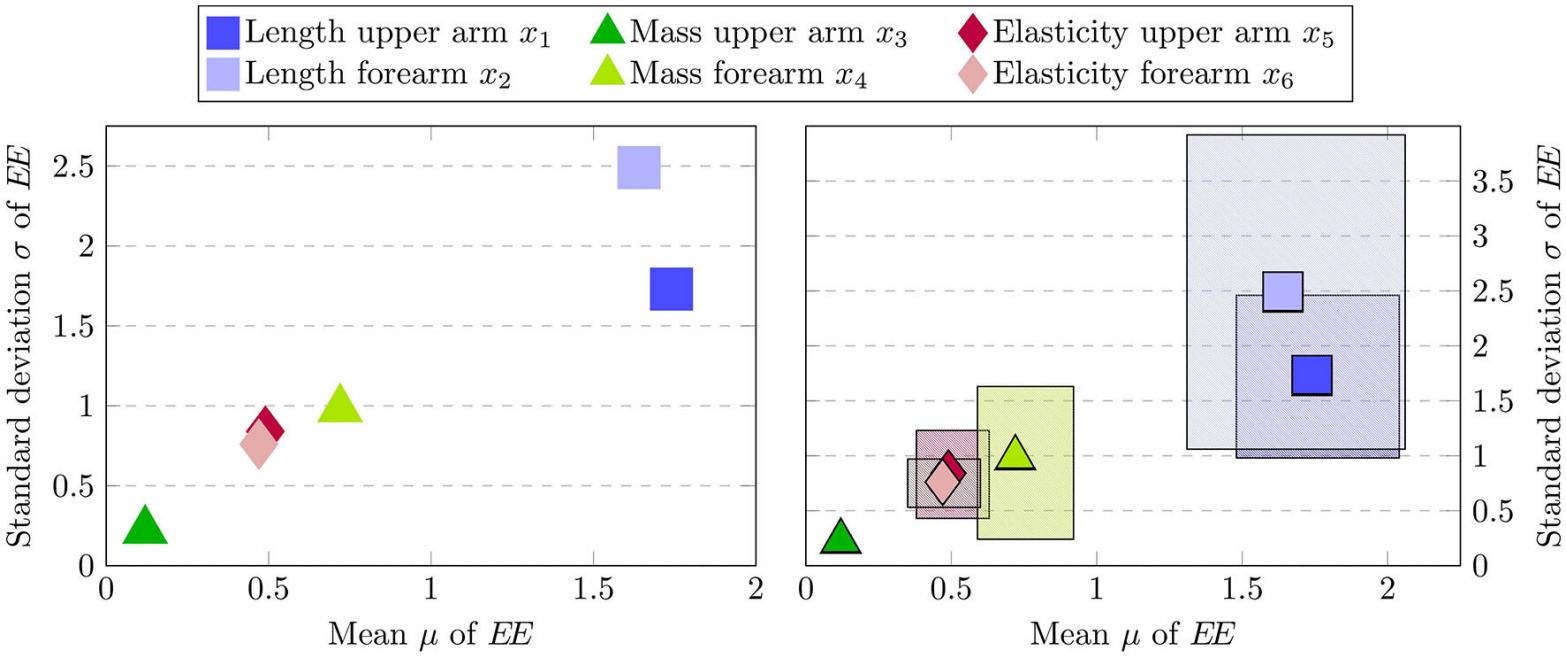
Estimated mean μ vs. standard deviation σ of the elementary effects *EE* **(left)** and approximated 95% confidence bounds via bootstrapping **(right)**. Here, each input factor is represented by one marker and the confidence bounds are represented by the patterned area associated with each marker.

The results of the elementary effect test are depicted in Figure 8. On the left-hand side, it is clearly visible that the estimated sensitivity measures indicate the mass of the upper arm *x*_3_ as the least influential input parameter. The low value estimated for both the mean and standard deviation implies that the input factor has both little direct impact on the estimated joint stiffness during the automated assessment procedure and moreover does not interact strongly with the remaining parameters. This makes sense intuitively since the mass of the upper arm is not expected to influence the estimated torque on the elbow level. However, due to the design of the passive mobilization experiment in Section 2.3, it is first necessary to drive the human arm into the desired initial configuration to start the procedure. Thereby different upper arm mass parameterization could potentially influence the precise starting state, which in turn can lead to slight changes in the estimated stiffness. However, from the results of the elementary effect test it is apparent that these disturbances do not impair the assessment process. Differently, the length of the upper and forearm exhibit the highest sensitivity both with respect to the mean and standard deviations. Therefore, the elementary effect method identifies the parameters associated with uncertainties due to kinematic incompatibilities as the most dominant ones. Lastly, the remaining parameters regarding the soft-tissue contact dynamics and the mass of the forearm are estimated to have a comparable sensitivity measure with the mass having a slightly larger impact in both μ and σ.

Sampling-based sensitivity analysis methods inherently approximate the true sensitivity indices given the observed samples. Therefore, especially when working with small to medium sample sizes, it is pertinent to validate the robustness of the obtained results. In order to investigate this, an additional robustness analysis can be performed (Pianosi et al., 2016) which assesses whether similar sensitivity measures would have been obtained with different input samples. This can be achieved in a sample-efficient manner by approximately computing the confidence bounds of the estimated similarity measures using bootstrapping (Efron and Tibshirani, 1993). Note that while bootstrapping is an efficient technique, the obtained confidence intervals do not constitute theoretically guaranteed bounds in general and can result in overly optimistic estimates when applied to the Morris method (Yang, 2011; Romano and Shaikh, 2012). However, applying the method still allows to retrieve valuable insights regarding the estimated sensitivity indices. The results of the robustness analysis are displayed in Figure 8 on the right. Here, a total of 300 μ and σ values are computed for each input factor, where each value is generated by drawing 150 samples with replacement from the original 1050 sampling points. Notably, the confidence bounds for the upper arm mass *x*_3_ are very small, thereby indicating that the mass of the upper arm can confidently be regarded as a non-influential input factor that can be fixed for subsequent analysis. In contrast, the upper arm length *x*_1_ and forearm length *x*_2_, which are identified as the most important ones by the elementary effect test, are associated with large confidence intervals. In particular the forearm length *x*_2_ features the highest uncertainty in the estimated sensitivity measures. Therefore, the results are not conclusive to make reliable statements beyond the screening of the upper arm mass and the deployment of further sensitivity analysis methods is required.

### 3.2. Factor prioritization using variance-based sensitivity analysis

Following the elementary effect test in the previous evaluation, we perform an additional variance-based sensitivity analysis to obtain a more rigorous understanding of the uncertain sensitivity patterns present in the human-exoskeleton system. To this end we exploit the findings of the prior section to fix the upper arm mass *x*_3_, as it is identified as a non-influential factor, which leads to a reduction of the computational load of the proposed variance-based analysis. For the input sample generation of the remaining parameter we use the two-phase sample procedure proposed for the variance-based approximation of the first-order and total-order indices (Saltelli et al., 2010). In the first phase, a total of 2*N* random samples is generated, which are referred to as base samples. Subsequently, *KN* additional input samples are produced by resampling vectors of the base samples. Thereby, this method requires *N*(*K* + 2) model evaluation for the estimation of the first-order and total-order effects and is computationally more efficient than a naive approach (Saltelli et al., 2010). Here, we set *N* = 3, 000 and investigate *K* = 5 input factors leading to a total of 21, 000 simulation runs. The random base samples are again obtained using the Latin hypercube method assuming a uniform distribution over the input parameters.

**Figure 9:**
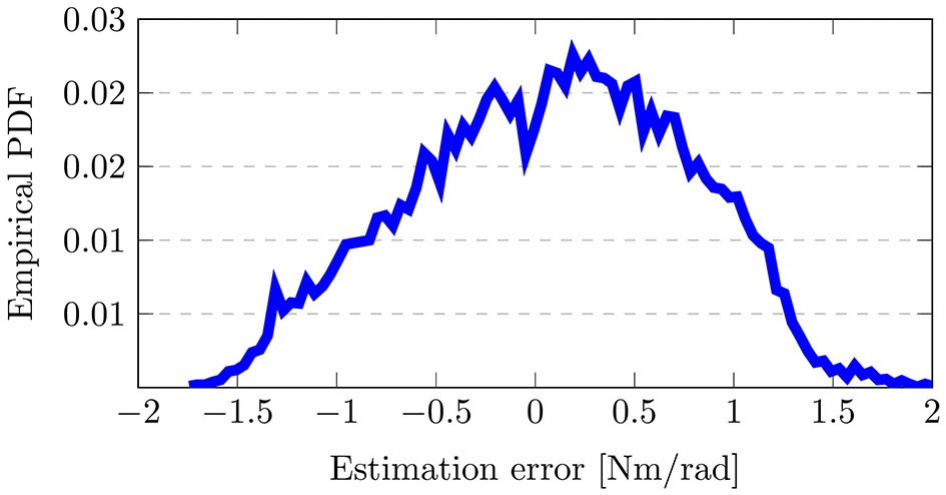
Visualization of the output distribution, i.e., error in the impedance parameter estimation, due to the sampled input parameters. Here, the empirical probability distribution function (PDF) is shown.

The resulting output distributions is shown in Figure 9 with the empirical probability distribution function (PDF), which is approximated from the output samples. Here, the output distribution, i.e., the estimation error in ***K***_*h*_, resembles a Normal distribution with a mean estimation error slightly larger than 0Nm/rad. Thereby, it can be seen how the sampling of uncertainties in the input variability space induces an output distribution and impacts the assessment results. Note that an implicit assumption in variance-based sensitivity analysis is that variance is an appropriate measure to capture uncertainty (Pianosi et al., 2016). Since the empirical PDF in Figure 9 resembles a Normal distribution and is neither multi-model nor highly-skewed, this assumption holds true, thus strengthening the viability of deploying the approach here.

**Figure 10:**
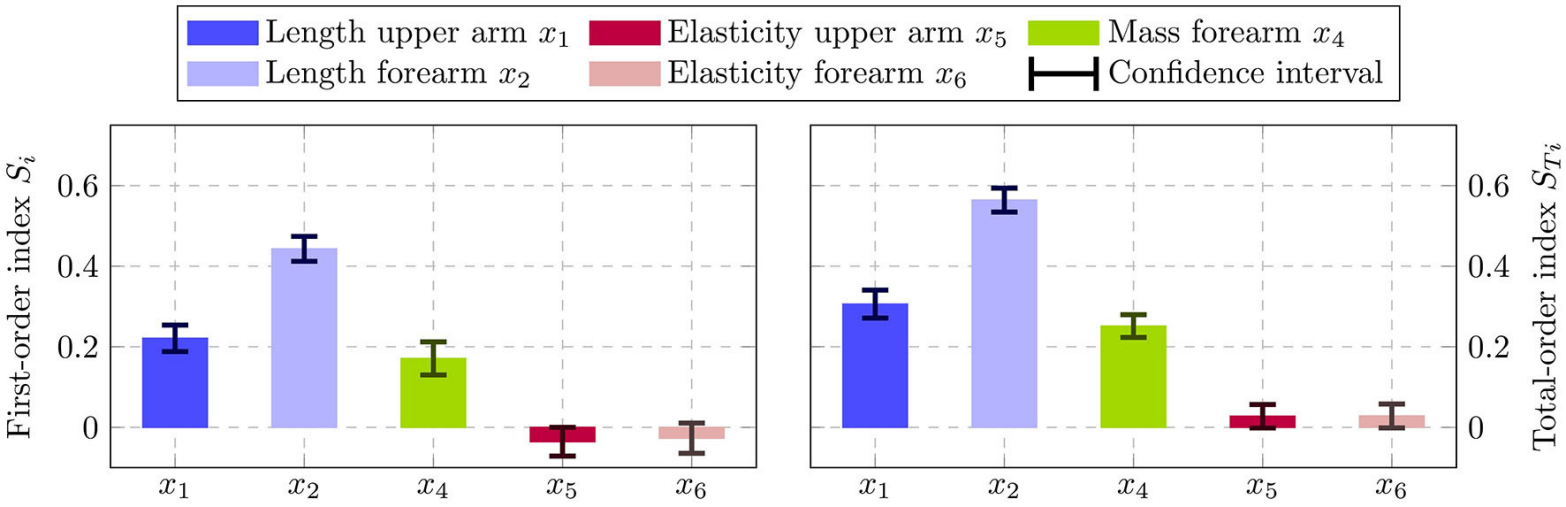
Estimated first-order indices *S*_*i*_ **(left)** and total-order indices *S*_*Ti*_ **(right)** with 90% confidence intervals using the variance-based sensitivity analysis. The left figure shows the most influential factor is *x*_2_ followed by *x*_1_ and *x*_4_. The total-order effects on the right identify both *x*_5_ and *x*_6_ to have no impact, since *S*_*Ti*_ = 0 constitutes a necessary and sufficient condition.

Figure 10 depicts the resulting first-order indices *S*_*i*_ on the left and total-order indices *S*_*Ti*_ on the right. Additionally, the 90% confidence interval are shown by the error bars, which are computed using bootstrapping. From the first-order effects it is clearly visible that the factors *x*_1_, *x*_2_ and *x*_3_ are the most influential ones, with the length of the forearm *x*_2_ having the highest impact. Moreover, the results indicate that the softness of the upper and forearm *x*_5_ and *x*_6_ are negligible, since their respective total-order indices are close to zero. Note that a total-order index of value zero constitutes a necessary and sufficient condition for an input factor to be non-influential (Pianosi et al., 2016). The negative signs for the first-order indices of *x*_5_ and *x*_6_ can be attributed to numerical errors, which are known to occur for input factors with negligible sensitivity indices when using the deployed sampling method (Saltelli et al., 2008). Moreover, the sum of the first-order effects computes to 0.78, while the sum of the total-order effects is 1.13. Since both sums are not equal to 1, it can be concluded that there are interaction effects present among the input factors in the system. Additionally, it can be seen in Figure 10 that the total-order indices of each factor are greater than the respective first-order indices. Thus, it can be inferred that all of the studied input parameter participate in the interactions.

**Figure 11:**
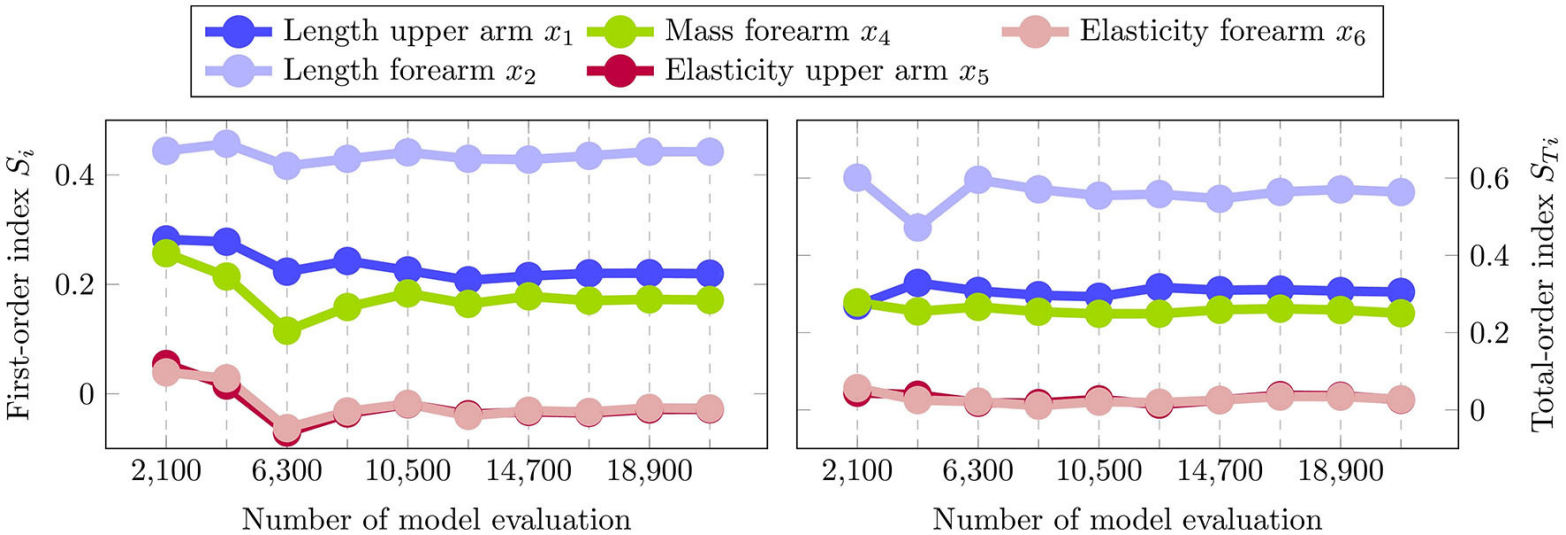
Convergence plot illustrating the estimated sensitivity indices using an increasing amount of sub-samples. Both the first-order and total-order indices converge quickly, which implies that a sufficient sample size is chosen for the variance-based sensitivity analysis.

Finally, we perform a convergence analysis to affirm the reliability of the obtained results. Since the sensitivity indices are approximated from samples, a convergence analysis assesses whether the evaluated sample size is sufficiently large to make a statement regarding the importance of the input factors. This can be done efficiently by recomputing the results from increasing sets of sub-samples of the original data set and analyzing the convergence of the observed indices (Nossent et al., 2011; Pianosi et al., 2016). The results of the performed convergence analysis are shown in Figure 11. Here it can be seen that both the first and total-order indices converge quickly when increasing the size of the sub-samples with few changes in the indices after sub-samples of half the size of the original set. This indicates that a sufficiently large input sample size is chosen in the evaluation. Since the error bars in Figure 10 are also small when compared to the estimated indices, the obtained results can be deemed robust. Therefore, we can conclude that the length of the forearm is the most influential source of uncertainty, with the upper arm length and the mass of the forearm following as the next most important factors.

### 3.3. Qualitative sensitivity analysis

In previous sections, we have analyzed the impact of uncertainties on the human-exoskeleton interaction from a quantitative manner, which is a particularly suitable approach when screening for influential and non-influential factors and when ranking those. By applying the elementary effect test and variance-based sensitivity analysis in Sections 3.1 and Section 3.2, input parameters associated with kinematic incompatibilities and erroneous dynamics model are identified as the most relevant uncertainties. However, little information regarding their functional influence on the system is retrieved and, while interaction between the inputs is indicated, their exact nature remains unclear. Therefore, we perform an additional qualitative sensitivity analysis to gain further insights into the most influential sources of uncertainty.

**Figure 12:**
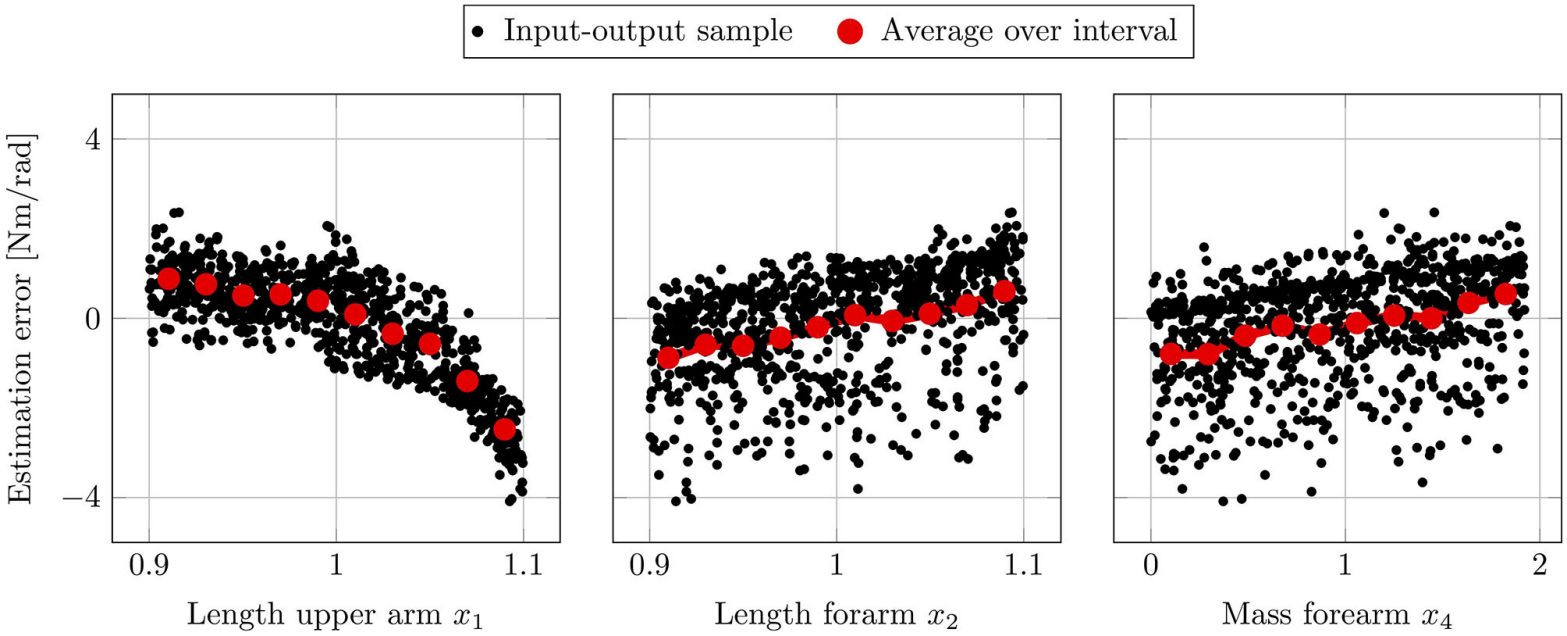
Scatter plot visualizing the output samples against input samples for variations of the upper arm length *x*_1_ **(left)**, variations of the forearm length *x*_2_ **(middle)** and variations of the forearm mass *x*_4_ **(right)**. The red dots illustrate the mean output for equidistant bins along each input.

Figure 12 visualizes the relationship between input and output samples for *x*_1_, *x*_2_, and *x*_4_. Each black dot in the scatter plot indicates an input-output sample pair, while the larger red dots depict the average output values over an interval range of the respective input. Here, equidistant intervals that split the input value ranges into 10 bins are used, which result in a width of 0.02 for *x*_1_ and *x*_2_, and 0.17 for *x*_4_. For the evaluation, a total of 1,500 input samples are generated assuming a uniform distribution for each parameter. Note that here the *x*_2_ sample range is slightly larger compared to the previous evaluation, since the sampling strategy of the qualitative sensitivity analysis is more robust to erroneous model responses, which can occur due to simulation failures caused by unreasonable input parameter combinations. In Figure 12 it is clearly visible that variation in the length of the upper arm *x*_1_ induce a nonlinear change in the output, while both forearm length changes *x*_2_ and forearm mass changes *x*_4_ have a linear influence. The linear relationship in *x*_2_ and *x*_4_ is consistent with the physical intuition for the examined system, since the gravitational component of the human arm dynamics in (1) is a linear function in the link length and the mass. Thus, it is indicated that the forearm length *x*_2_ has to be considered as a source of uncertainty with respect to both kinematic incompatibilities and modeling errors, which leads to a better understanding of the high sensitivity ranking of *x*_2_ in the variance-based analysis. Differently, the output exhibits a nonlinear behavior in *x*_1_ with a continuous decrease in the slope for larger upper arm lengths. Thereby, it can be derived that beyond a certain threshold the misalignment in the center of rotations due to variations in *x*_1_, lead to extreme errors in the output value and may cause catastrophic failures. Thus, despite the relative lower prioritization in Section 3.2, the upper arm length remains a significant uncertainty and it needs to be ensured that the mismatch to its nominal values is below certain runaway boundary conditions.

**Figure 13:**
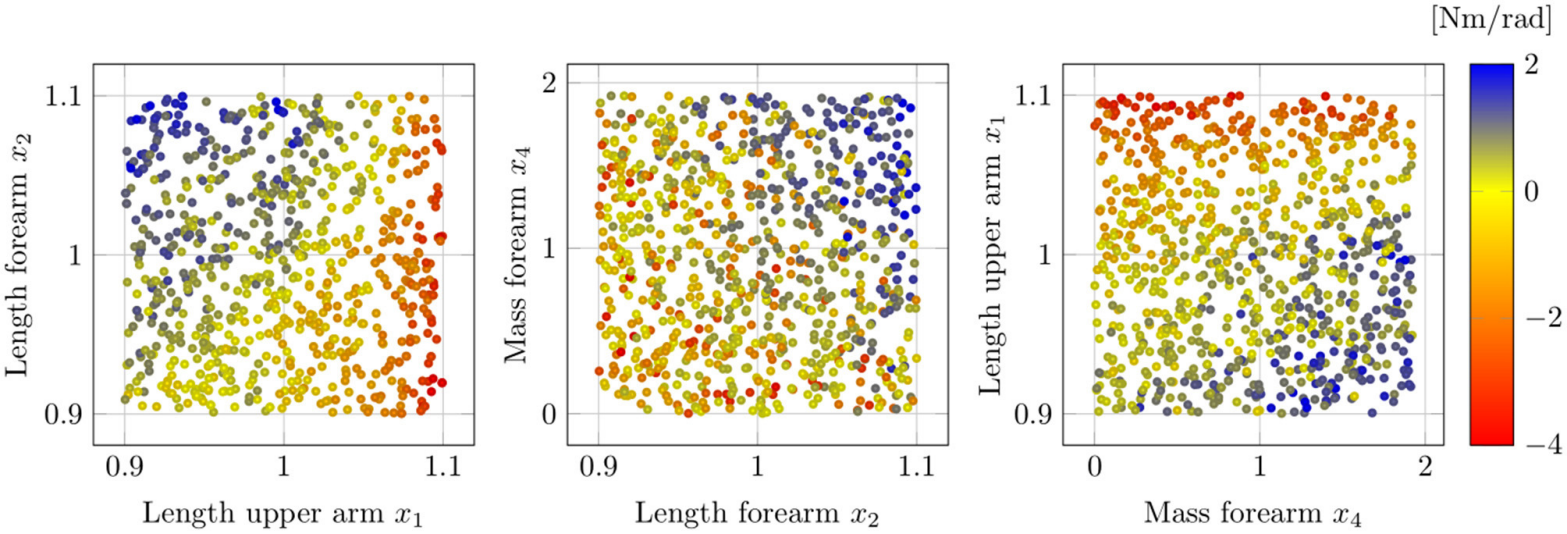
Colored scatter plot depicting samples of the *i*-th input parameter on the *x*-axis against the *j*-th on the *y*-axis, where the marker color indicates the respective estimation error. By observing emerging pattern in the plot, conclusions regarding the interaction of two input factors can be derived.

Finally, we visualize the interaction between the input parameters using colored scatter plots in Figure 13, where one input factor is depicted on the *x*-axis against another one on the *y*-axis with the marker color indicating the output value. Here, the emergence of patterns provides an indication for the interaction between two factors. From Figure 13 on the far right it can be seen that little interaction is taking place between upper arm length *x*_1_ and forearm mass *x*_4_, since the output values do not change significantly with concurrent changes in the input parameters. However, it can be detected that the upper arm length *x*_1_ is dominant for very large values, since the markers along the maximal y-axis values are all colored in red. On the other hand, a slight interaction between the forearm length *x*_2_ and mass *x*_4_ can be inferred from the middle plot, where the estimation error appears to grow strongly, if both input parameters are increased jointly. Intuitively, this can be ascribed to the fact that an increase in the forearm length also shifts the center of mass of the link, which in turn increases the influence of the forearm mass. Lastly, in Figure 13 on the left it can clearly be seen that for very high values of *x*_1_ the upper arm length dominates the output, which is indicated by the red marker coloring along maximal x-axis values.

## 4. Discussion

The present study performed a quantitative sensitivity analysis of the major sources of uncertainty present in an upper-limb human-exoskeleton system, and their impacts on the arm impedance parameter estimation was investigated. The performed analysis indicates kinematic incompatibilities and errors in the nominal dynamics model as the most influential sources of uncertainty. Specifically, variations in the assumed forearm length belong to both classes of uncertainty and appear to be the most significant factor according to the results in Figure 10. However, given a wider input variability space, the influence due to variations in the upper arm length dominates, as shown in the qualitative analysis in Figures 12, 13. Here, the results indicate that for slight kinematic misalignments within a 5% range of the nominal upper arm length, the resulting estimation error only grows approximately linearly. However, when the upper arm misalignment increases beyond the approximately linear range, the nonlinear functional behavior results in a blow up of the estimation error. While qualitative sensitivity analysis approaches are more ambiguous, this finding makes sense intuitively, as the upper arm length is associated with offsets in the center of rotation, which is typically considered a significant source of uncertainty (Schiele, 2008; Jarrassé and Morel, 2012). In addition to the above-described link lengths, the mass of the forearm is the third-most relevant source of uncertainty according to both the elementary effect test and the variance-based sensitivity analysis. Here, the forearm mass has implications regarding the nominal dynamics model, since it is relevant for both the gravitational and inertial properties of the human arm. In contrast, the contact dynamics due to soft-tissue at the attachment are the least relevant as the results in Figure 10 indicate them to be non-influential.

Given the results, it can be seen that uncertainty has a significant effect on the exoskeleton-based arm impedance estimation. In order to help reduce overconfidence in assessment results, the estimation procedure may benefit from employing uncertainty-aware regression techniques, e.g., Gaussian Processes, which model uncertainty explicitly, and thus make it transparent for the clinician (Rasmussen and Williams, 2005). Besides modeling the uncertainty, practical steps can be taken to increase the precision of the assessment by exploiting insights provided by our sensitivity analysis. In particular, reducing the effect of kinematic incompatibilities should be prioritized here. More specifically, a close alignment of the center of rotations has to be ensured. Inclusion of passive DoFs on the shoulder as well as the elbow level can mitigate the influence of kinematic incompatibilities (e.g., Vitiello et al., 2013). Additionally, special care should be taken during the donning procedure to ensure an ideal alignment before and during the usage. Second, our sensitivity analysis shows that errors in the nominal dynamics model, due to inaccuracies in the modeling of gravitational and inertial properties of the human arm, adversely affect the impedance estimation result. Therefore, measures should be taken to reduce these effects. This can be achieved by performing more extensive identification procedures for the human arm model instead of relying on standardized models derived from anthropometric data. The benefits of deploying more personalized models has been demonstrated recently in rehabilitation scenarios (Just et al., 2020). While modeling inaccuracies are expected to be less prevalent for the robotic system, they may also adversely affect the assessment. For example in scenarios where unknown and nonlinear friction components influence the robot joints (Chang et al., 2009), the device dynamics may differ from the original identification. Therefore, ensuring the accuracy of the robot model also needs to be considered in practice when performing automated assessment.

The simulation environment proposed in the presented study emulates realistic load transmissions between the human and exoskeleton via a mechanical interface composed of supporting cuffs and straps. In addition, we facilitate soft contacts by augmenting the human musculoskeletal model by simulated soft-tissue at the attachment areas. To the best of the authors' knowledge, it is the first upper-limb human-exoskeleton simulation that acknowledges the contact dynamics at the mechanical interface between human and robot by implementing both the interface and the human soft-tissue explicitly. Therefore we believe that the developed high-fidelity simulation platform lends itself well for exploitation in diverse use cases and is particularly suitable to investigate safety and ergonomics in control development. The consideration of ergonomics in physical human-robot interaction is a field that has recently gained growing attention and is considered crucial for driving advances in human-robot collaboration (Gualtieri et al., 2021; Sunesson et al., 2023). Having an explicit implementation of the physical interface is particularly relevant here, in order to accurately represent loads arising at the human limb during interaction with an exoskeleton. Moreover, our proposed simulation platform also provides utility in assisting simulation-based hardware development of wearable robotics, as the consideration of safety and ergonomics is desirable here (Agarwal et al., 2010).

While the present study quantitatively analyzed how uncertainties in the human-exoskeleton interaction impact the arm impedance estimation, some simplifying assumptions were made. First, an idealized, fully known robotic system is assumed. Despite the fact that inertial and gravitational components can reasonably be derived for the exoskeleton, commonly, unknown friction dynamics remain. However, we do not expect this to be a significant issue, since a multitude of friction compensation strategies exist (Huang et al., 2019), which can straight-forwardly be applied in the considered scenario. Another assumption was made with respect to the simulation of spastic behavior of the human arm. In particular, we did not consider joint synergies or phase-dependent descriptions of spasticity. Since in this work the focus lied on isolating the influence of uncertainties on the mechanical interaction and consequently on the assessment, the consideration of a more complex spasticity model would provide limited additional benefit to the objective of the study. Still the presented human musculoskeletal simulation allows for the inclusion of different spasticity behaviors in principle. Thus, despite these limitations, the presented results enable us to derive the most relevant sources of uncertainty that impact the physical human-exoskeleton interaction, and thereby help increase the precision of exoskeleton-based arm impedance estimation.

## 5. Conclusion

We conclude that this work presents a novel framework to analyze the influence of sources of uncertainty in the human-exoskeleton interaction and their impact on the exoskeleton-based impedance estimation. Due to an increasing demand for robot-based neurorehabilitation and assessment, we argue that the explicit consideration and quantification of uncertainties is paramount, as this allows for more robust and trustworthy estimates. To this end, a human-exoskeleton simulation environment is developed to facilitate the use of sampling-based sensitivity analysis methods. The performed sensitivity analysis indicates that uncertainties significantly impact the impedance estimation, and are primarily caused due to kinematic incompatibilities and inaccuracies in the nominal rigid body dynamics model of the human arm. Therefore, the findings of the study may also be used to increase the precision of exoskeleton-based automated assessment, i.e., by extending model calibrations of the human arm, more careful donning procedures or by deploying uncertainty-aware regression techniques. In the future, we plan to exploit this framework to develop approaches for uncertainty reduction during exoskeleton-based impedance estimation, in order to reduce the estimation uncertainty below pre-defined tolerances. Thus, providing a constructive approach for improving exoskeleton-based automated assessment.

## Data availability statement

The original contributions presented in the study are included in the article, further inquiries can be directed to the corresponding author.

## Author contributions

ST and RS implemented the simulation, validated the components with experimental data, and wrote the first draft of the manuscript. ST performed the sensitivity analysis. SE and SH provided the critical revisions. All authors contributed to the conceptualization, methodology, read, and approved the manuscript.

## Footnotes

^1^Open-source code of the upper-limb human-exoskeleton simulation environment is available at: https://github.com/stesfazgi/rehyb_mujoco.
